# Unveiling Van Der Waals Forces and Associated Hamaker Constants Across the Nanomaterial Landscape With Advanced Atomic Force Microscopy

**DOI:** 10.1002/smtd.70898

**Published:** 2026-08-11

**Authors:** Amir Farokh Payam, Horacio V. Guzman, Alexandre Tkatchenko

**Affiliations:** ^1^ Nanotechnology and Integrated Bioengineering Centre School of Engineering Ulster University Newtownabbey UK; ^2^ Institut de Ciència de Materials de Barcelona ICMAB‐CSIC, Carrer dels Til.lers, Cerdanyola del Vallès Bellaterra Spain; ^3^ Department of Physics and Materials Science University of Luxembourg Luxembourg City Luxembourg

**Keywords:** hamaker constant, intermolecular force, nanomaterials, nanoscopic scale, nanotechnology, van der waals force

## Abstract

Van der Waals (vdW) forces and the associated Hamaker constants underpin a broad spectrum of phenomena in biology, chemistry, materials science, and nanotechnology, shaping processes from protein folding to the stability of two‐dimensional (2D) materials. Achieving precise nanoscale quantification of these interactions remains a challenge, as conventional approaches lack the spatial resolution to resolve localized vdW effects in complex systems. Atomic force microscopy (AFM), with its nanometer sharp probe, has overcome many of these limitations, enabling high‐resolution and nondestructive measurement of vdW forces and the Hamaker constants. Here, the physics of vdW interactions and recent advances in AFM methodologies that have transformed their quantification is reviewed. Applications span 2D materials, where AFM reveals layer–layer and layer–substrate interactions critical for device performance, and biological systems, where vdW forces govern protein assembly, viral mutation, and cellular transformations. Emerging integration of AFM with molecular and theoretical frameworks for intermolecular interactions based on quantum mechanics further expand the frontier, offering unprecedented accuracy and insights into nanoscale phenomena. Perspectives and prospects of AFM‐based vdW force detection is discussed, highlighting its potential in providing a high‐resolution and noninvasive quantitative instrument for the development of novel applications in materials science, molecular biology, and quantum technologies.

## Graphical abstract

Van der Waals interactions are reviewed through the lens of advanced atomic force microscopy, highlighting recent progress in nanoscale quantification of Hamaker constants. Emerging methodologies enable high‐resolution mapping of intermolecular forces in 2D materials and biological systems, bridging experimental measurements with quantum‐informed models and offering new insights into complex nanoscale phenomena.

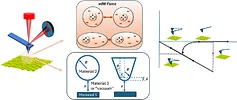

## 1 Introduction

The van der Waals (vdW) force—a fundamental interaction arising from instantaneous and induced electric dipoles in atoms or molecules—is a subtle yet pervasive force that profoundly impacts molecular and atomic behavior. Definitions of vdW forces vary in different fields of research. Intimately connected with the vdW equation of state, in chemistry, vdW interactions encompass both attractive dispersion interactions at long interatomic distances and Pauli repulsion when atomic electron densities overlap. Within physics, vdW force encompasses attractive—or also repulsive when retardation of light is involved—forces beyond simple covalent or ionic bonds, and they are essential for phenomena as varied as gas condensation, surface adhesion, and molecular recognition [1, 2, 3]. The Hamaker constant, a measure of the vdW interaction strength between materials, quantifies this force at nanoscale separations, providing a critical parameter for understanding material stability and behavior at small scales [4].

These vdW interactions and the Hamaker constant are central to numerous fields. In biology, they govern essential molecular processes such as protein folding, molecular docking, and cellular adhesion [5, 6, 7], playing a vital role in cellular signaling, immune responses, and drug efficacy [7, 8, 9, 10]. In physics and chemistry, vdW forces shape atomic and molecular structures, influencing gas behaviors and surface energies [11, 12]. In materials science and nanotechnology, they are crucial for adhesion, cohesion, and surface interactions [4, 13], affecting material manipulation, nanocomposite engineering, and the assembly of nanoscale devices [14, 15].

Various methods exist to quantify vdW forces and the Hamaker constant, including surface force apparatus [4, 16, 17, 18, 19], immersion calorimetry [20], dynamic light scattering [21], and ellipsometry [22]. Surface force apparatus offers detailed force–distance measurements but is limited by invasive procedures and spatial resolution [4, 16, 17, 18, 19], while immersion calorimetry provides bulk interaction insights, though with less spatial resolution [23]. Dynamic light scattering captures information in solution‐based systems but often averages over nanoscale heterogeneities [24]. On the other hand, ellipsometry's limitation lies in its sensitivity to surface roughness, material properties, and environmental factors, which can introduce inaccuracies in the results [24, 25]. While each technique has its advantages, these methods generally face challenges in capturing localized vdW interactions at the nanoscale, restricting their utility in high‐spatial precision applications.

Complementary experimental approaches such as atom scattering and diffraction techniques provide direct access to gas–surface interaction potentials and vdW forces [26, 27, 28]. Early gas–surface diffraction studies enabled the determination of interaction potentials and long‐range dispersion coefficients (e.g., *C*_3_) through analysis of diffracted beam intensities and bound‐state resonances [26]. More recent developments extend these approaches to complex systems, including topological insulators and semimetals, providing insights into weak physisorption regimes, interaction lifetimes, and atom–surface dynamics [27]. In addition, heavy atom diffraction has been used to extract vdW potentials with high accuracy and to probe surface corrugation effects, offering complementary information to scanning probe techniques [28]. While each method offers distinct advantages, these approaches generally face limitations in spatial localization or experimental complexity, restricting their utility for high‐resolution nanoscale mapping of vdW interactions.

Atomic force microscopy (AFM) techniques have thus emerged as a powerful approach for vdW force measurements, particularly valuable in probing the nanoscale interactions of 2D materials and vdW heterostructures [24, 25, 29]. AFM methods enable precise, localized measurement of vdW forces through interactions between the AFM tip and sample surface, providing unparalleled spatial resolution and sensitivity [24, 25, 30, 31].

**FIGURE 1:**
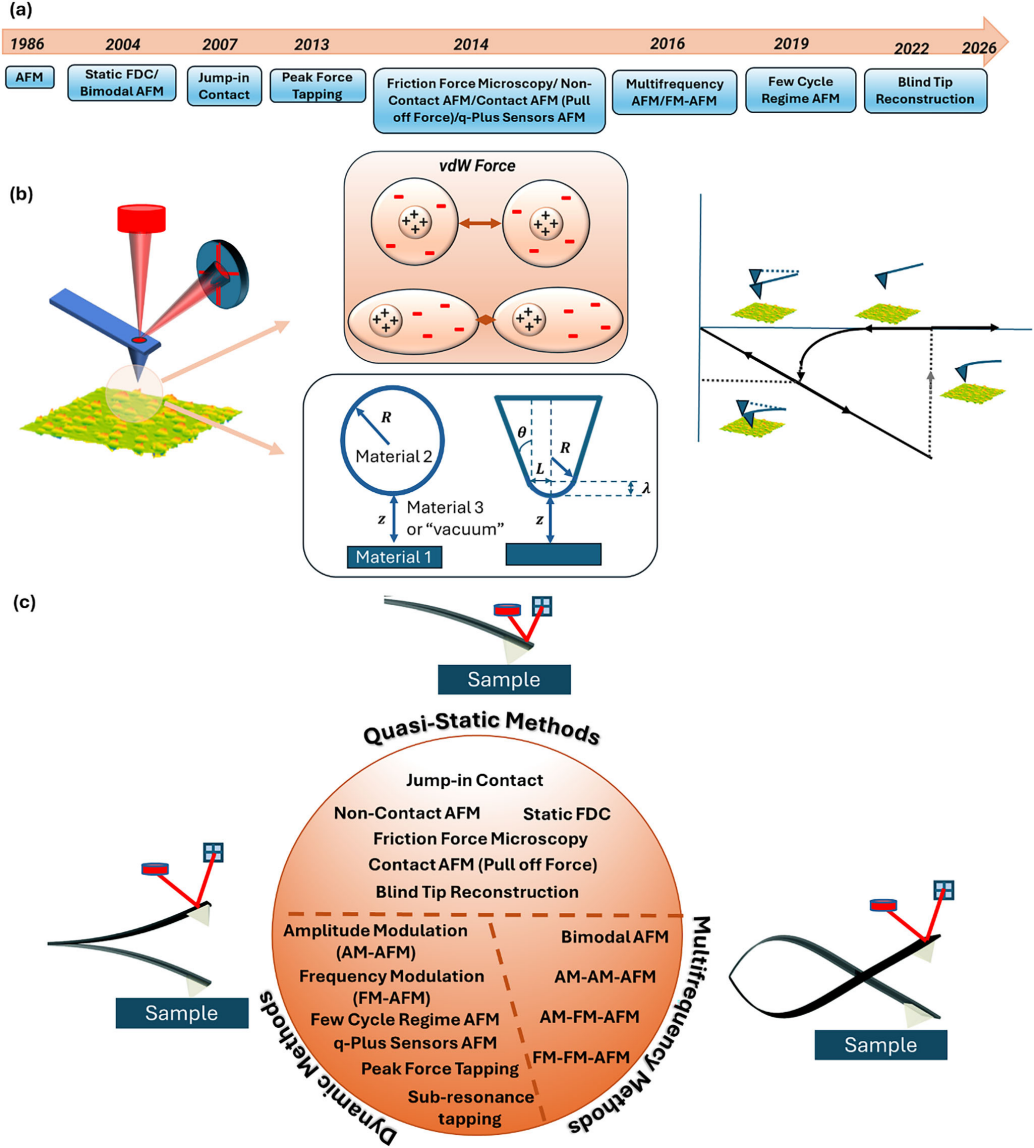
Schematic representation of AFM‐based methods for probing vdW interactions and evaluating the Hamaker constant. (a) A historical timeline illustrating the key developments in atomic force microscopy that enabled quantitative measurement of the Hamaker constant and vdW forces, highlighting the evolution from early force–distance measurements to advanced multifrequency approaches. (b) Conceptual schematic of AFM operation in the context of vdW interaction, showing how the AFM tip and cantilever interact with the sample surface at different tip–sample separations. The schematic depicts the nature of the vdW potential, the distance‐dependent interaction mechanism, the role of tip geometry in modeling the vdW force and the contribution of the cantilever tip ensemble to the overall force signal. In the distance‐dependent mechanism, the cantilever tip starts far from the surface where vdW forces are negligible. As the tip approaches, increasing vdW attraction causes the cantilever to bend toward the surface. Once the attraction exceeds the cantilever's restoring force, the tip snaps into contact. On retraction, the tip remains in contact until the restoring force pulls it free. (c) Classification of AFM methodologies used for detecting vdW interactions and determining the Hamaker constant, broadly categorized into quasi‐static techniques (e.g., force–distance curves), dynamic techniques (e.g., frequency shift and amplitude modulation), and multifrequency approaches that exploit multiple resonance channels for improved resolution and sensitivity. This integrative overview underscores the progression of AFM strategies and their relevance in accurately characterizing fundamental nanoscale interactions.

This review presents an in‐depth analysis of recent advancements in AFM‐based techniques and theoretical approaches for quantifying vdW forces and the associated Hamaker constants (Figure 1). Through this comprehensive exploration, we underscore the essential role of vdW forces in modern scientific fields and the evolving methodologies to measure and manipulate these forces effectively, particularly in the context of 2D and biological materials.

In this review, we will provide a discussion and perspective on this field about bridging AFM data and fundamental physical vdW interactions, a question that was raised since AFM invention [32] and has not found an easy answer yet. Here, we stress that the interpretation of experimental and theoretical work is complex, and that the connection between theoretical development and experimental measurements in specific vdW‐puzzles is often required. Finally, we outline future directions where AFM may unlock further insights into nanoscale forces, advancing material science and device engineering.

## 2 Theoretical Modeling of vdW Force and Hamaker Constants for AFM Techniques

The quantum mechanical origin of vdW forces was first established by London, Casimir, and Lifshitz [4, 33] describing interactions between atoms and macroscopic bodies. In AFM, these forces are typically modeled either through continuum approaches based on Lifshitz theory or through semiempirical atomistic descriptions [34, 35]. At the nanoscale, vdW potentials are often approximated as a sum of pairwise additive dispersion terms of the form *C*_6_/*z* ^6^ derived from quantum mechanical principles [31]. However, first‐principles calculations remain computationally expensive due to the complexity of electron correlation effects [36], and density functional theory (DFT) with dispersion corrections is commonly employed, albeit with empirical approximations [4].

A complete description of molecule–surface interactions requires accounting for both exchange and correlation energies [31]. At large separations, where wavefunction overlap vanishes, exchange contributions disappear, and correlation can be treated perturbatively. In this regime, the Lifshitz–Zaremba–Kohn theory yields an asymptotic potential [37]:

(1) $$V\left(z\right)∼-\frac{C_{3}}{z^{3}}-\frac{C_{4}}{z^{4}}-\frac{C_{5}}{z^{5}}-\cdots$$

The leading term is usually approximated as [31]

(2) $$V_{a-s}\;\left(z\right)=-C_{3}{\left(z-z_{0}\right)}^{-3}$$

with [31]

(3) $$C_{3}^{A,S}=\frac{\hbar }{4\pi }\int^{\infty }du\frac{\varepsilon_{S}\left(iu\right)-1}{\varepsilon_{S}\left(iu\right)+1}\alpha_{A_{i}}\left(iu\right)$$

where ε_*S*_(*iu*) is the substrate dielectric function, $\alpha_{A_{i}}\left(iu\right)$ is the polarizability of atom *A_i_*, and *z*_0_ is the vdW reference plane.

The vdW potential between an atomically sharp AFM tip and a sample surface is derived from the atom–atom interaction law via integration over the continuous half‐space, leading to the well‐known atom–surface *C*_3_/*z* ^3^ dependence for large separations. For realistic AFM tips, the vdW interaction is commonly approximated using a sphere–flat geometry, resulting in a force law as the derivative of the interaction potential proportional to *C_H_*/*z* ^2^ where *C_H_* is the Hamaker constant that characterizes the material‐dependent vdW interaction strength and *z* is the distance between the AFM tip and the sample surface.

For an AFM tip modeled as a sphere (material “2”) interacting with a flat substrate (material “1”) (Figure 1b), the force–distance dependence is rigorously given by [38]

(4) $$F_{ts}\;\left(C_{H},z\right)=-\frac{2C_{H}R^{3}}{3z^{2}{\left(z+2R\right)}^{2}}$$

where *C_H_* = *C*_*H*12_ ​ is the Hamaker constant. If one assumes that the tip *R* ≫ *z*, the force simplifies to [38, 39]

(5) $$F_{ts}\;\left(C_{H},z\right)=-\frac{C_{H}R}{6z^{2}}$$

For the case that the tip involves a truncated pyramid with a spherical cap, which leads to a different force–distance dependence [39, 40],

(6) $$\begin{array}{ccc} F_{ts}\;\left(C_{H},z\right) & = & -\frac{C_{H}}{6}\left[\frac{\lambda^{3}\left(3Rz+R\lambda -z\lambda \right)}{z^{2}{\left(z+\lambda \right)}^{3}}+\frac{L^{2}}{{\left(z+\lambda \right)}^{3}}\right.\\ & & \left.+\;\frac{4\tan\theta }{\pi }\left(\frac{L+\tan\theta \left(z+\lambda \right)}{{\left(z+\lambda \right)}^{2}}\right)\right] \end{array}$$

where the geometric parameters *R*,  *L*,  λ, and θ are defined in Figure 1b.

Using the Lifshitz theory, the Hamaker constant can be derived from dielectric functions of the interacting media. For example, the conductor–dielectric in vacuum or air [41]

(7) $$C_{Hcd}\approx \frac{3h}{8\sqrt{2}}\frac{c_{1}\upsilon_{e,c}\upsilon_{e,d}}{c_{2}\left(c_{2}\upsilon_{e,d}+\upsilon_{e,c}\right)}$$

Equation (7) is a simplified version of the Lifshitz theory for vdW interactions of an isotropic Drude conductor (*c*) and a dielectric (*d*) across vacuum or air, where $c_{1}\equiv n_{d}^{2}-1$​ and $c_{2}\equiv {\left(n_{d}^{2}+1\right)}^{1/2}$, *n_d_* is the refractive index of the dielectric, *h* is Planck's constant, $\upsilon_{e,d}$​ is the absorption frequency of the dielectric, and $\upsilon_{e,c}$ is the plasma frequency of the conductor.

The Drude model provides an analytical description of the dielectric response of a conductor and therefore enables an analytical approximation of vdW interactions involving metallic systems. It should be noted, however, that vdW interactions are not limited to conductors, and they also arise between the dielectric and insulating materials. In such cases, more general dielectric response models must be considered, with the Drude approximation representing a convenient and physically insightful limiting case for metal–dielectric interactions.

Dielectric–dielectric interactions in a medium is described by [42, 43]

(8) $$\begin{array}{ccc} & & C_{H132}\approx \frac{3}{4}k_{B}T\left(\frac{\varepsilon_{1}-\varepsilon_{3}}{\varepsilon_{1}+\varepsilon_{3}}\right)\left(\frac{\varepsilon_{2}-\varepsilon_{3}}{\varepsilon_{2}+\varepsilon_{3}}\right)\\ & & +\frac{3h\upsilon_{e}}{8\sqrt{2}}\frac{\left(n_{1}^{2}-n_{3}^{2}\right)\left(n_{2}^{2}-n_{3}^{2}\right)}{\sqrt{n_{1}^{2}+n_{3}^{2}}\sqrt{n_{2}^{2}+n_{3}^{2}\left\{\sqrt{n_{1}^{2}+n_{3}^{2}}+\sqrt{n_{2}^{2}+n_{3}^{2}}\right\}}} \end{array}$$

where the permittivity ε_*i*_ (*i* = 1, 2, 3) and refractive indices *n_i_* are indexed according to the materials. Also, *k_B_* is the Boltzmann constant, *T* is the temperature, $\upsilon_{e}$ is single absorption frequency, and *h* is the Plank's constant.

Importantly, the microscopic coefficient *C*_3_​ and the macroscopic Hamaker constant *C_H_* ​ are linked but not equivalent: while *C*_3_​ ​ describes single atom–surface interactions, *C_H_* ​ arises from integrating these contributions over macroscopic bodies. Bridging this gap requires improved theoretical treatments. Intermolecular perturbation theory (IPT) [44] such as SAPT refines pairwise summation by perturbatively accounting for electron correlation and polarization screening, whereas many‐body dispersion (MBD) theory [44] captures collective fluctuation effects that dominate at larger length scales, providing a more accurate route from atomic polarizabilities to bulk Hamaker constants. These advances are critical in AFM, where vdW forces dominate tip–sample interactions, influencing adhesion, force spectroscopy, and high‐resolution imaging. By combining experimental AFM data with updated frameworks such as Lifshitz theory, IPT, and MBD, researchers can extract more reliable Hamaker constants, refine vdW models, and improve nanoscale characterization of both soft and hard materials.

Interactions between realistic materials arise from a wide range of contributions and couplings. For example, electrostatics, polarization, and dispersion interactions can couple rather strongly and lead to unconventional distance‐dependent power laws and screening/anti‐screening phenomena. Full theory of intermolecular interactions for complex materials does not exist yet, and we must proceed carefully in understanding how different effects couple constructively and destructively in a given system of interest. This complexity can be illustrated by numerous examples in the literature, including Faraday‐cage effects at the nanoscale [45, 46], control of vdW interactions via applied static charges [47], or inhomogeneous electric fields created by vdW interactions between extended molecules [48, 49].

## 3 Motivation

The significance of vdW forces and the Hamaker constant in advanced materials science and biological systems cannot be overstated [50, 51, 52]. These forces govern the nanoscale interactions that are fundamental to the stability and functionality of numerous systems. In biological contexts, vdW forces contribute to protein stability, folding, and interactions with cell membranes, processes critical for cellular function, signaling, and immune response [7, 8, 9, 10, 50, 53].

**FIGURE 2:**
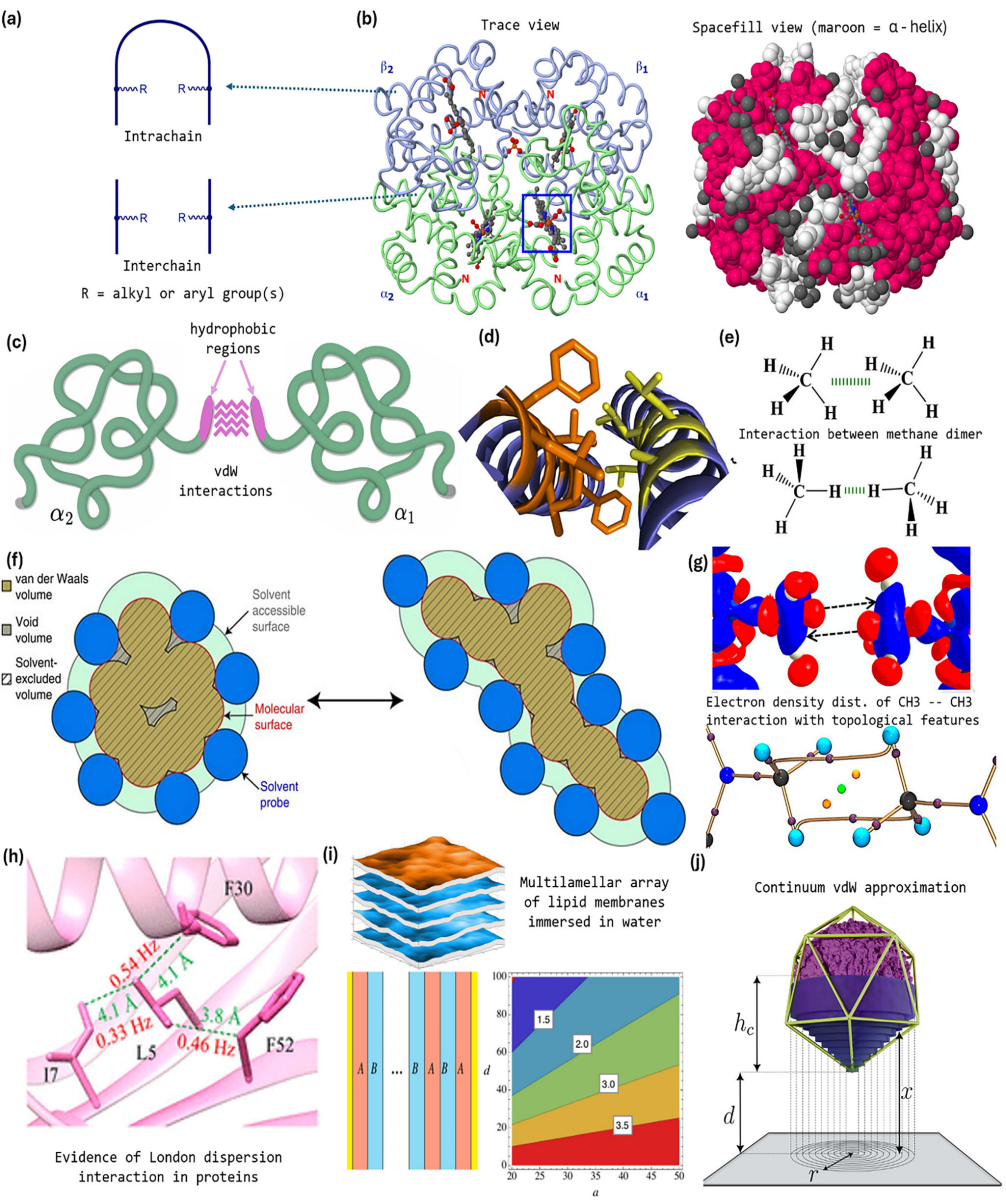
Examples of vdW interactions and their roles in protein structure and biological applications. (a) Many functional proteins depend more on their tertiary structure than on regular secondary motifs. Myoglobin illustrates this: although ∼70% α‐helical, its activity relies on a compact globular fold stabilized by side‐chain interactions (disulfide, electrostatic, hydrogen bonding, and vdW). Polar residues face outward, nonpolar residues form the core with intrachain and interchain interactions, and bends are introduced by residues such as proline, isoleucine, and serine. The figure is reproduced with permission from reference [55]. (b) vdW interactions between nonpolar side chains (e.g., Phe, Tyr, Gly, Ala, Val, Leu, Ile) stabilize tertiary folding by promoting hydrophobic associations in aqueous environments. The figure is reproduced with permission from reference [55]. (c) Quaternary structure arises when folded subunits assemble into oligomeric complexes. Hemoglobin, for example, consists of two α‐chains and two β‐chains, each with a heme group embedded in a hydrophobic pocket, where the exclusion of water prevents the oxidation of Fe^2^^+^. vdW interactions between hydrophobic regions of subunits further stabilize quaternary assemblies. (d) Interdigitation of bulky side chains in helices 7 (orange) and 8 (yellow) of the *E. coli* J‐type co‐chaperone Hsc20 (PDB 1fpo) generates extensive vdW contacts while burying hydrophobic residues from the solvent, illustrating the synergy of vdW forces and the hydrophobic effect. The figure is reproduced with permission from reference [56]. Copyright 2010, Springer Nature. (e) London dispersion interactions in methane dimers adopt two preferred geometries with favorable C···C distances of 4.2 Å (top) and 5.0 Å (bottom). The figure is reproduced with permission from reference [57]. Copyright 2023, American Chemical Society. (f) Molecular surface representation showing the solvent‐excluded volume (*V*_SE_, shaded), defined using a 1.4 Å probe (blue spheres). *V*_SE_ comprises van der Waals volume (*V*_vdW_, dark yellow) and internal void volume (*V*_Void_, gray). Upon unfolding, solvent exposure increases and buried voids become accessible. The figure is reproduced with permission from reference [59]. Copyright 2017, Springer Nature. (g) Experimental electron density deformation map (top) and molecular graph (bottom) showing bond paths and critical points for CH_3_···CH_3_ interactions; red regions indicate charge depletion, blue regions charge accumulation. Adapted from reference [57]. Copyright 2023, American Chemical Society. (h) Observation of vdW‐mediated scalar coupling (vdW JCC) in NMR experiments on nonpolar residues of protein GB3 (PDB 1IGD), arising from CH···CH London dispersion interactions (adopted with values from reference [57]. Copyright 2023, American Chemical Society. (i) Multilamellar array of lipid membranes immersed in water, on the top a representation of a membrane with fluctuating positions, bottom left illustrates a model system with a rigid array of alternating solvent (B) membrane (A) and solvent (B) regions. The bottom right shows the Hamaker coefficient, *H*(*a*, *d*) in (zJ) as a function of *d* separation between membranes, and *a* is the thickness of the lipid bilayers in nm. The figure is adopted and reproduced with permission from reference [73]. (j) The Derjaguin approximation has been used to quantify the vdW forces at the continuum level for diverse geometries, commonly depending on the distance *x*; however, in biology, the shapes are much richer and specific than a sphere. Here an icosahedral symmetry shows that the vdW force will also depend on the three folding symmetries, shown the fivefold viral symmetry with analytical Derjaguin solution for a given *hc* cutoff.

For example, vdW forces help stabilize α‐helices and β‐sheets, which are essential in the structure of enzymes and antibodies [50, 54]. They play a crucial role in stabilizing protein structures by contributing to folding and maintaining integrity at various organizational levels. In the primary structure, vdW interactions influence the conformation of the polypeptide chain. In secondary structures such as α‐helices and β‐sheets, these forces help stabilize backbone interactions. At the tertiary level, vdW interactions between side chains, especially in aromatic residues, shape the protein's three‐dimensional (2D) form [55] (Figure 2a). In quaternary structures, they aid in maintaining protein complexes by stabilizing subunit interactions. vdW interactions between exterior hydrophobic parts of the contributing subunits help to maintain the quaternary structure of a protein aggregate in water [55] (Figure 2b,c).

An illustrative example of these principles can be found in the *Escherichia coli* (*E. coli*) J‐type co‐chaperone HSC20 (PDB 1fpo), where short atom–atom distances enable extensive vdW contacts to stabilize tertiary packing (Figure 2d) [56]. Specifically, helices 7 (orange) and 8 (yellow) interdigitate through bulky, branched side chains, forming a large interhelical contact area in an antiparallel coiled coil (PDB 1fpo) [56]. These close contacts not only enhance stability through vdW interactions but also bury hydrophobic side chains away from the solvent, demonstrating the cooperative interplay between vdW forces and the hydrophobic effect in maintaining protein structure.

To further illustrate the significance of vdW interactions in protein structure and function, recent experimental and computational studies highlight the role of London dispersion forces in stabilizing protein conformations. For instance, methane dimer models (Figure 2e) reveal the preferred distances at which vdW interactions optimize molecular packing, a principle that extends to biomolecular assemblies [57]. Moreover, charge redistribution in CH···HC interactions (Figure 2e) demonstrates how vdW forces modulate intra‐ and intermolecular stability in both small alkanes and larger biomolecules [57]. Experimental 3D deformation density maps (Figure 2e) further provide direct visualization of these interactions, reinforcing their contribution to molecular cohesion [57].

Furthermore, vdW forces play a crucial role in protein folding by influencing the volume and packing of protein structures (Figure 2f) [58, 59]. The total volume of a protein in a solution consists of its vdW volume, void volume and hydration effects. Compared to the unfolded state, intramolecular hydrogen bonding reduces the vdW volume in the native state. However, this difference is offset by hydration effects upon unfolding, maintaining the overall volume balance (Figure 2f) [59]. Advanced computational methods, such as ProteinVolume [60], accurately estimate these volume components. While electrostatic effects contribute minimally to volume changes, vdW interactions remain fundamental in stabilizing protein structure by optimizing atomic packing [59]. Moreover, electrostatic effects of proteins interacting with materials like Silica are shown to be highly localized [61, 62].

Quantifying vdW interactions within proteins or between proteins and cell membranes can uncover molecular recognition mechanisms, providing critical insights for drug design, where vdW interactions largely dictate the binding affinity and stability of drug–protein complexes, influencing therapeutic efficacy and specificity [10, 63, 64].

Notably, nucleic magnetic resonance (NMR)‐based detection of vdW couplings in protein GB3 (Figure 2g) confirms the presence of dispersion interactions between nonpolar residues, underscoring their critical role in tertiary and quaternary protein structures [57]. Such findings not only enhance our understanding of protein folding mechanisms but also inform rational drug design by elucidating how vdW interactions influence binding specificity and molecular recognition. At continuum length scales, the Hamaker constant for the multilamellar array of lipid membranes (Figure 2h) in water can be estimated. The essential component of a membrane is the lipid bilayer—a planar layer of finite thickness composed of a hydrocarbon core with hydrophilic boundaries facing the aqueous solution [65]. vdW interactions between lipid membranes were, in fact, the first example of using full Lifshitz theory in condensed media [66].

In addition, vdW forces are thought to play an essential role in virus mutation and cell transformation (Figure 2i,j), as subtle changes in vdW interactions can affect viral protein folding and the stability of virus capsids [63, 64, 67, 68, 69, 70]. These forces can impact the viral entry process and mutation pathways, as mutations that alter the surface interactions of viral proteins can enable evasion from host immune responses or improve binding to host cells, as seen in highly mutable viruses [63, 67, 69]. In cell transformation, such as the shift from a normal to a cancerous state, changes in vdW interactions can contribute to alterations in cell membrane adhesion, enabling cancer cells to break free from primary tumor sites and invade other tissues [71, 72]. Investigating vdW interactions in these contexts can thus inform both therapeutic interventions and diagnostic strategies for viral and cancer biology.

**FIGURE 3:**
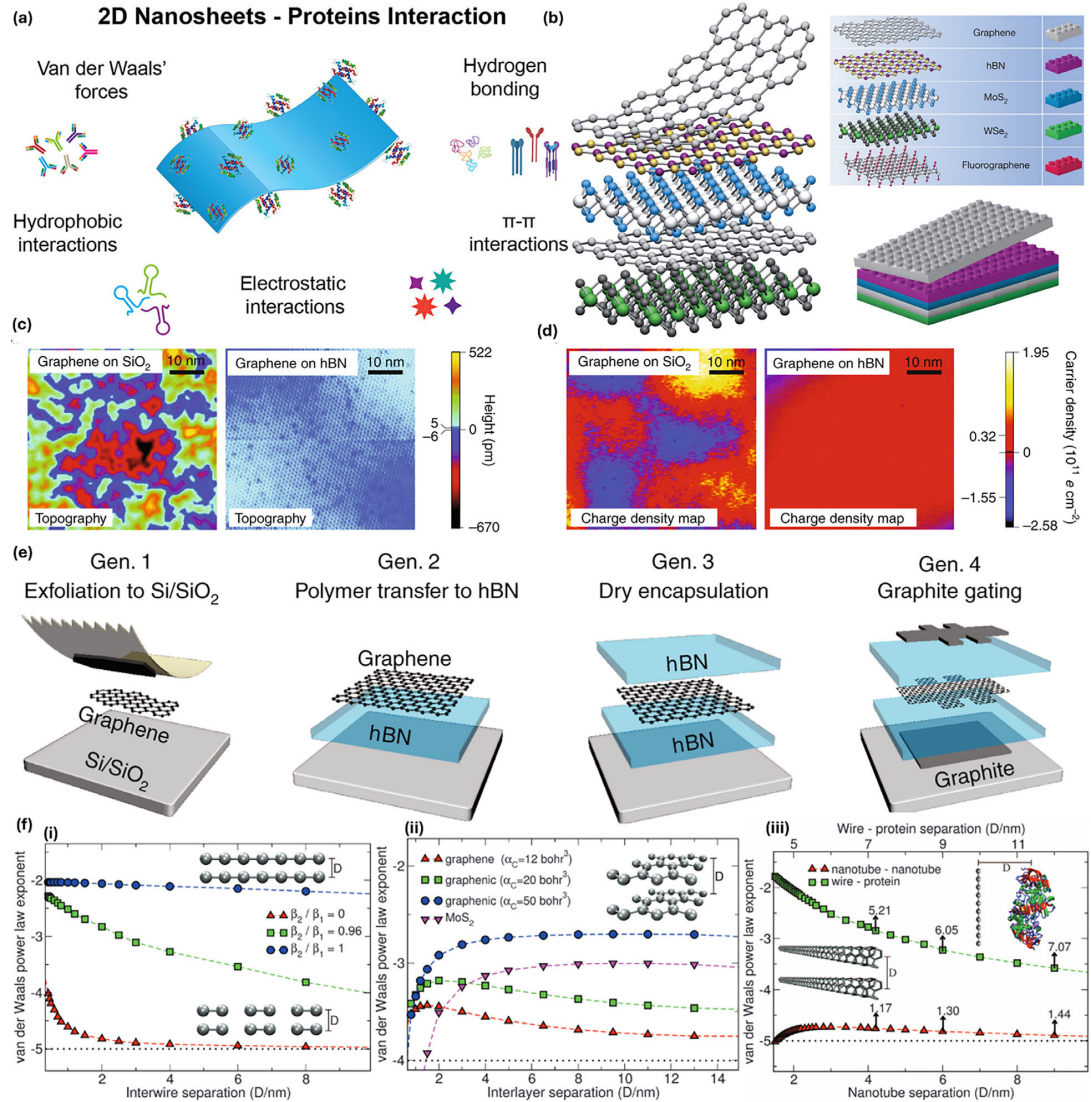
Overview of vdW interactions and their role in low‐dimensional materials and device fabrication. (a) Schematic of two‐dimensional interactions between nanosheets and proteins, including vdW forces, hydrophobic interactions, electrostatic interactions, π–π stacking, and hydrogen bonding [6]. The figure is reproduced with permission from reference [6]. Copyright 2023, American Chemical Society. (b) Construction of vdW heterostructures. Analogous to Lego blocks (right panel), 2D crystals can be assembled into a wide range of layered structures. While conceptually similar to molecular beam epitaxy, this “atomic Lego” approach follows different assembly rules and employs distinct materials. The figure is reproduced with permission from reference [81]. Copyright 2013, Springer Nature. (c, d) Comparison of STM topography (c) and charge‐density maps (d) for graphene on hBN versus graphene on SiO_2_. The figure is reproduced with permission from reference [51]. Copyright 2019, Springer Nature. (e) Evolution of device fabrication techniques: (generation 1) mechanical exfoliation of graphene onto Si/SiO_2_; (generation 2) graphene transfer onto hBN using a polymer film; (generation 3) dry encapsulation in hBN via vdW assembly; (generation 4) hBN encapsulation with graphite gates, the figure is reproduced with permission from reference [51]. Copyright 2019, Springer Nature. (f) vdW interactions across low‐dimensional systems. (i) Tight‐binding model of interwire vdW interactions: interwire interaction energy exponents for parallel 1D carbyne‐like atomic wires at separation *D*, considering insulating (*b*_2_/*b*_1_ = 0), metallic (*b*_2_/*b*_1_ = 1), and intermediate (*b*_2_/*b*_1_ = 0.96) cases with *d*_C–C_ = 2.0 Å. Strong deviations from the asymptotic *D* ^−^^5^ law (dotted line) are observed, consistent with MBD interactions [44]. (ii) Power laws for interlayer vdW interactions: interlayer exponents for 2D graphene layers and MoS_2_ at varying polarizabilities ($\alpha_{C}^{0}\;$= 12–50 bohr^3^) computed using the MBD model. Substantial deviations from the *D* ^−^^4^ law predicted by pairwise‐additive approaches (dotted line) are found [44]. (iii) Power laws for vdW interactions between complex nanostructures: MBD exponents for two parallel (3, 3) carbon nanotubes and a wire–protein nanostructure (insets) as a function of center‐of‐mass separation *D*. As with 1D and 2D systems, significant deviations from the asymptotic *D* ^−^^5^ behavior are observed. The ratio of MBD to pairwise interaction energies ($\Delta E_{\mathrm{vdW}}^{\mathrm{LR}}$/$\Delta E_{\mathrm{vdW}}^{\mathrm{LR}(2)}$) increases with *D*, reflecting the nontrivial coupling between delocalized charge‐density fluctuations in these nanostructures. The figure is reproduced with permission from reference [44]. Copyright 2016, Science.

These biological examples highlight vdW interactions as a unifying physical principle that governs molecular recognition, structural stability, and functional adaptation in living systems. Extending this concept to engineered platforms, 2D materials offer atomically defined, vdW‐dominated surfaces that can deliberately interface with proteins and other biomolecules, and hence influence their size, charge, shape, and biological identity (Figure 3a) [6]. These short‐range forces arise from electron cloud interactions and require a large contact area, making them essential for protein adsorption and nanosheet stacking. Graphene's π‐electron‐rich surface supports strong vdW and cation–π interactions, while transition metal dichalcogenides (TMDs) like MoS_2_ exhibit even stronger vdW forces due to higher surface charge densities. In Xenes such as selenene, vdW interactions dominate due to their unique atomic architecture, enabling effective binding with biomolecules.

In biosensor technology, quantifying and optimizing vdW interactions are essential for enhancing diagnostic sensitivity and accuracy. For instance, a recent study demonstrated that optimizing vdW interactions within nanophotonic biosensors based on low‐dimensional vdW materials enables highly sensitive, real‐time in vivo measurements [74]. Such advancements are pivotal for point‐of‐care diagnostics, where sensors must achieve a delicate balance of sensitivity and specificity to detect biomolecular interactions with high precision [75, 76]. Furthermore, vdW materials facilitate the integration of electronic and photonic components within sensors, yielding multifunctional, hybrid biosensing platforms [74, 77]. Recent findings also show that vdW materials enhance surface‐enhanced infrared absorption (SEIRA) sensors, allowing real‐time analysis of biochemical reactions with unparalleled sensitivity [74, 78]. These innovations promise to transform early disease diagnosis and continuous health monitoring.

In materials science, vdW interactions play a pivotal role in the structural engineering and stability of 2D polymorphic materials such as graphene, hexagonal boron nitride (hBN), and TMDs [51, 52, 78]. These forces are essential for maintaining the integrity of 2D materials, as they govern both the interlayer bonding and the interactions between layers and substrates [6, 79, 80]. For instance, Figure 3b illustrates how weak vdW forces can hold together complex layered assemblies with atomic precision while maintaining structural stability [81].

Building on this foundation, research has rapidly advanced toward the design of vdW heterostructures, where different 2D crystals are vertically stacked to form artificial materials with tailored properties. This modular approach, often likened to atomic‐scale Lego assembly, allows precise control over stacking order and interfacial characteristics. Within each monolayer, atoms are connected by strong covalent bonds, while adjacent layers are bound by vdW forces. As shown in Figure 3b, such a strategy has been recently realized experimentally and has enabled the fabrication of highly ordered, multilayer heterostructures with functional advantages that go beyond those of single‐layer systems [81].

Among these heterostructures, hBN has emerged as an outstanding substrate for graphene‐based devices, owing to its atomically flat surface and minimal charge inhomogeneity. Scanning tunneling microscopy (STM) topography and charge‐density maps (Figure 3c,d) demonstrate that graphene on hBN exhibits significantly reduced surface roughness (2.3 × 10^10^ cm^−2^ r.m.s.) compared with graphene on SiO_2_ (8.2 × 10^10^ cm^−2^ r.m.s.) [51]. This superior interfacial quality minimizes disorder, thereby enhancing the electronic performance of 2D devices [82, 83].

The progressive improvement of graphene device fabrication can be traced across four generations (Figure 3e), each reflecting a deeper exploitation of vdW interactions. First‐generation devices relied on the SiO_2_ substrate [52], but suffered from high disorder. Second‐generation devices introduced graphene–hBN stacks assembled using polymer transfer techniques [84], which reduced disorder but introduced polymer residues and limited multilayer integration. To address this, third‐generation devices adopted dry transfer methods that used vdW forces alone to assemble large‐area, hBN‐encapsulated graphene layers. This approach minimized contamination and eliminated polymer contact, resulting in bubble‐free interfaces and improved electrical performance [85], though some influence from the underlying substrate persisted. Finally, fourth‐generation devices refined this approach by incorporating graphite gates and contacts, eliminating electrostatic potential fluctuations from metal gates and achieving the lowest disorder levels reported to date [86]. Taken together, these developments illustrate how vdW interactions have evolved from being regarded as a passive bonding mechanism to becoming an enabling force for the atomic‐scale design of ultraclean, high‐performance 2D material systems.

Tuning vdW forces allows researchers to control layer adhesion, optimize interlayer conductivity, and manage mechanical properties—all essential for high‐performance applications in flexible electronics, optoelectronics, and energy storage [78]. For instance, the ability to control vdW interactions at phase boundaries in TMDs enables the precise adjustment of electronic properties, making them suitable for advanced transistors and photodetectors [51, 52, 78]. The Hamaker constant becomes crucial here, offering a quantitative measure to predict stability and layer‐to‐layer coherence, which is foundational in ensuring reliability and longevity in devices dependent on nanoscale stability and functionality. Theoretical advances have greatly expanded our understanding of vdW forces by moving beyond classical pairwise interactions [87, 88, 89], density‐functional theory [87], molecular dynamics (MD) simulations [89] to incorporate many‐body effects [90, 91] and quantum mechanical phenomena [92]. Studies on long‐range vdW forces between graphene and substrates, for instance, reveal that collective atomic wavelike deformations contribute to stress distribution over larger distances than previously understood, affecting wear and mechanical stability in composite materials [92]. This theoretical framework, which integrates quantum‐continuum approaches, is particularly relevant in tribology and membrane mechanics [93, 94], where a refined understanding of vdW interactions can enhance material durability.

Furthermore, recent research shows that nanostructured materials such as proteins, nanotubes, and 2D materials exhibit complex vdW interactions with nonlocal charge‐density fluctuations. These fluctuations result in distinct power‐law behaviors, altering interaction energies over nanoscale distances [44] (Figure 3f). For biologically relevant structures, such as phospholipid membranes, these wavelike density fluctuations create coherent vdW interactions that stabilize membrane adhesion and assembly [44]. Traditional models often fail to capture these nuances, highlighting the need for advanced methods to explore vdW interactions within complex biological and synthetic materials, potentially enabling the design of tunable vdW systems for enhanced adhesion and nanoscale assembly [44].

Recent theoretical developments directly address these limitations by establishing fully predictive, multiscale frameworks that unify atomistic electronic, and nuclear dynamics with continuum electromagnetic descriptions. These approaches account for geometry, short‐range electronic delocalization, dissipation, and collective nuclear vibrations (phonons), while simultaneously treating long‐range electromagnetic interactions in arbitrarily shaped environments [95]. Studies applying these frameworks to low‐dimensional carbon materials—such as fullerenes, carbyne, and graphene—show that phonon–photon coupling at nanometric separations generates delocalized phonon polaritons that significantly modify infrared response and vdW forces, including the emergence of nonmonotonic power laws and temperature‐dependent behaviors observable in experiments [95]. By rigorously coupling density‐functional‐level electronic fluctuations to shape‐dependent electromagnetic fields, these methods reveal orders‐of‐magnitude deviations from classical Casimir–Polder, nonretarded, or pairwise‐additive predictions, particularly at mesoscopic scales where molecular size, nonlocal excitations, and electromagnetic retardation strongly influence interaction energies [96]. Importantly, these general formulations demonstrate that the boundary between “atomistic” and “macroscopic” bodies is fundamentally arbitrary, enabling seamless computation of vdW free energies and radiative heat transfer across systems ranging from small biomolecules near metallic surfaces to extended graphene sheets [97]. These advances provide accurate and computationally efficient tools for exploring complex geometries and multi‐scale environments previously inaccessible to conventional vdW theories [96, 97].

The Hamaker constant, a central parameter in vdW interactions, is indispensable in optimizing interactions within these materials and applications [36]. Its accurate determination is essential in applications ranging from drug delivery to nanoscale energy devices. Lifshitz theory remains foundational for calculating the Hamaker constant across varied frequency ranges; yet limitations in available optical data for many materials pose challenges [36, 92, 93]. Experimental approaches that provide direct force–distance measurements have thus become invaluable. For example, research by Gobre and Tkatchenko on vdW scaling laws [36] in carbon‐based nanostructures demonstrates that vdW coefficients deviate from classical models due to multipole interactions and quantum effects. These insights stress the importance of accurate, high‐resolution measurements in understanding and applying vdW interactions in complex nanostructures [36, 98].

Given its adaptability, precision, and sensitivity to nanoscale forces, AFM has become crucial for quantifying vdW forces and the Hamaker constant [24, 31, 80, 99, 100, 101, 102]. AFM techniques provide nanoscale spatial resolution, offering unique insights into the force–distance dependence of vdW interactions in complex systems [24, 100, 101]. The following section reviews the latest advancements in AFM methodologies for vdW force measurement, emphasizing the synergy between theoretical models and AFM technology in delivering reliable, high‐resolution data for material science and biological applications. Through AFM, we can bridge theoretical advancements with experimental validation, enhancing our understanding and control of vdW interactions across a broad range of fields.

## 4 Methods

Various AFM‐based techniques have been developed to measure vdW forces, visualize vdW materials, and quantify the Hamaker constant, each with unique operational modes and excitation‐detection mechanisms. Quasi‐static methods, which involve recording force–distance curves, offer a straightforward approach to vdW force quantification, though they require extensive measurements and can be time‐consuming for mapping. Friction force microscopy has been applied to determine the Hamaker constant for materials such as graphite and MoS_2_, providing detailed insights into surface interactions [103]. Dynamic techniques, such as amplitude‐modulation AFM (AM‐AFM) or tapping‐mode AFM and frequency‐modulation AFM (FM‐AFM), deduce the Hamaker constant by fitting experimental data with theoretical models, enabling faster mapping and improved control in data acquisition [104, 105]. Bimodal AFM techniques further advance these capabilities by allowing simultaneous mapping of material properties and topography, although some numerical approximations may slow down property map generation [30, 100, 102, 106, 107]. Table 1 summarizes the applications of AFM techniques in quantifying the vdW force and Hamaker constant of diverse materials.

**TABLE 1:** Summary of Hamaker constants (H) measured by various AFM techniques across different materials, environments, and tip configurations. H quantifies vdW interaction strength (in zJ) between the tip and the sample.

Materials	AFM method	AFM tip	Measured H	Lifshitz/Calculated H	Other techniques	Refs.
HOPG (air)	Bimodal AFM	Si	16 zJ	132 zJ		[30]
HOPG (air)	Bimodal AFM	Si	129 zJ	132 zJ		[30]
HOPG (air)	NC‐AFM	Si_3_N_4_	155± 33 zJ			[39]
HOPG (vacuum)	IFA	SiO_2_	132 zJ	136 zJ		[103]
HOPG (air)	FM‐AFM	Si	160± 15 zJ	180± 25 zJ	125± 25 zJ	[25]
HOPG (nitrogen atmosphere with residual oxygen content below 0.2%)	Contact AFM	Si_3_N_4_	(4.9 ± 0.1) × *R* (10^−27^ Jm			[80]
HOPG (nitrogen atmosphere with residual oxygen content below 0.2%)	Contact AFM	Si_3_N_4_	(4 ± 0.03) × *R* (10^−27^ Jm			[80]
HOPG (water)	FM‐AFM	Si	6± 4 zJ			[25]
Mica (air)	Bimodal AFM	Si	13 zJ	80 zJ		[30]
Mica (air)	Bimodal AFM	Si	128 zJ	80 zJ		[30]
Mica (air)	FM‐AFM	Si	40± 10 zJ	40± 5 zJ	50± 10 zJ	[25]
Mica (air)	JC‐AFM	Si_3_N_4_	64 ± 7 zJ	128 zJ (vacuum); 24.5 zJ (water)	135 zJ (Vacuum); 22 zJ (water)	[24]
Calcite (air)	Bimodal AFM	Si	19 zJ	81 zJ		[30]
Calcite (air)	Bimodal AFM	Si	30 zJ	81 zJ		[30]
calcite (101̅4) plane (air)	Bimodal AFM	Si	30± 10 zJ			[102]
PFDA (air)	Bimodal AFM	Si	10 zJ			[30]
PFDA (air)	Bimodal AFM	Si	17 zJ			[30]
Graphene suspended (monolayer) 1LG (air)	Bimodal AFM	Si	75± 8 zJ			[130]
2LG (air)	Bimodal AFM	Si	209 ± 61 zJ			[130]
Multilayers‐G (air)	Bimodal AFM	Si	269 ± 128 zJ			[130]
Graphene (monolayer) on Cu (1LG/Cu) (air)	Bimodal AFM	Si	171 ± 38 zJ	152.2 zJ		[130]
2LG/Cu (air)	Bimodal AFM	Si	246 ± 41 zJ	217.1 zJ		[130]
Multilayers‐G (air)	Bimodal AFM	Si	360 ± 123 zJ	268.7 zJ		[130]
Graphene (monolayer) on SiO_2_ (1LG/SiO_2_) (air)	Bimodal AFM	Si	59 ± 12 zJ	41.6 zJ		[130]
2LG/SiO_2_ (air)	Bimodal AFM	Si	80 ± 31 zJ	165.8 zJ		[130]
Multilayers‐G/SiO_2_ (air)	Bimodal AFM	Si	213 ± 162 zJ	239.2 zJ		[130]
1LG/ SiO_2_ (nitrogen atmosphere with residual oxygen content below 0.2%)	Contact AFM	Si_3_N_4_	(7.5 ± 0.4) × *R* (10^−27^ Jm			[80]
2LG/ SiO2 (nitrogen atmosphere with residual oxygen content below 0.2%)	Contact AFM	Si_3_N_4_	(5.4 ± 0.5) × *R* (10^−27^ Jm			[80]
3LG/SiO2 (nitrogen atmosphere with residual oxygen content below 0.2%)	Contact AFM	Si_3_N_4_	(5 ± 0.2) × *R* (10^−27^ Jm			[80]
Monolayer Fluorinated Graphene on SiO2 (1LFG/SiO2) (nitrogen atmosphere with residual oxygen content below 0.2%)	Contact AFM	Si_3_N_4_	(0.0 ± 0.7) × *R* (10^−27^ Jm			[80]
1LG/1LfG/SiO_2_ (nitrogen atmosphere with residual oxygen content below 0.2%)	Contact AFM	Si_3_N_4_	(22.2 ± 1.6) × *R* (10^−27^ Jm			[80]
Al_2_O_3_ (vacuum)	BTR‐AFM	Si	150 zJ		170 zJ	[114]
α‐ Al_2_O_3_ (air)	NC‐AFM	Si_3_N_4_	147 ± 34 zJ	148.5 ± 3.5 zJ		[39]
SiO_2_ on Si substrate (vacuum)	BTR‐AFM	Si	130 zJ		120 zJ	[112]
SiO_2_ (air)	JC‐AFM	Si_3_N_4_	66 ± 27 zJ	121 zJ (vacuum); 20.7 zJ (water)	55 ± 5 zJ	[24]
SiO_2_ (nitrogen atmosphere with residual oxygen content below 0.2%)	Contact AFM	Si_3_N_4_	(0.9 ± 0.2) × *R* (10^−27^ Jm			[80]
SiO_2_ (nitrogen atmosphere with residual oxygen content below 0.2%)	Contact AFM	Si_3_N_4_	(8.2 ± 0.3) × *R* (10^−27^ Jm			[80]
MoS_2_ (vacuum)	IFA	SiO_2_	132 zJ	136 zJ		[103]
MoS_2_ (vacuum)	IFA	MoS_2_	280± 20 zJ	270 zJ		[103]
MoS_2_ (air)	FM‐AFM	Si	100± 20 zJ	200± 100 zJ	105 ± 10 zJ	[25]
MoS_2_ (nitrogen atmosphere with residual oxygen content below 0.2%)	Contact AFM	Si_3_N_4_	(5.1 ± 0.1) × *R* (10^−27^ Jm			[80]
1L MoS_2_/ SiO_2_ (nitrogen atmosphere with residual oxygen content below 0.2%)	Contact AFM	Si_3_N_4_	(6.3 ± 0.6) × *R* (10^−27^ Jm			[80]
2L MoS_2_/ SiO_2_ (nitrogen atmosphere with residual oxygen content below 0.2%)	Contact AFM	Si_3_N_4_	(6.5 ± 0.5) × *R* (10^−27^ Jm			[80]
1LMoS_2_/ Au (air)	Bimodal AFM	Si	∼ 80 zJ			[100]
2LMoS_2_/ Au (air)	Bimodal AFM	Si	∼ 100 zJ			[100]
Gold (Au) (air)	Bimodal AFM	Si	∼ 40 zJ			[100]
CaF_2_ (vacuum)	IFA	CaF_2_	71 zJ	70 zJ		[103]
Amorphous Silica (air)	NC‐AFM	Si_3_N_4_	64 ± 15 zJ	65.5 ± 0.5 zJ		[39]
PS (air)	NC‐AFM	Si_3_N_4_	85± 29 zJ	75 ± 4 zJ		[39]
PS (air)	FM‐AFM	Ti/Ir	55± 10 zJ	65 ± 5 zJ		[25]
PS (air)	AM‐AFM	Si	130 ± 70 zJ		145 ± 15 zJ	[99]
PMMA (air)	NC‐AFM	Si_3_N_4_	96 ± 27 zJ			[39]
PMMA (air)	FM‐AFM	Ti/Ir	60 ± 10 zJ	65 ± 5 zJ		[25]
ABS Polymer (air)	NC‐AFM	Si_3_N_4_	74 ± 29 zJ			[39]
LDPE (air)	AM‐AFM	Si	115 ± 35 zJ		160 zJ	[99]
DNA on mica (air)	Bimodal AFM	Si	20 ± 5 zJ			[102]
Single Spike α *variant* *of SARS−CoV−2* (air)	AM‐AFM	Si	18 ± 7 zJ			[119]
Single Spike β *variant* *of SARS−CoV−2* (air)	AM‐AFM	Si	20 ± 5 zJ			[119]
Single Spike γ *variant* *of SARS−CoV−2* (air)	AM‐AFM	Si	10 ± 4 zJ			[119]
hBN (air)	FM‐AFM	Si	105 ± 50 zJ	110 ± 10 zJ		[25]
MoTe_2_ (air)	FM‐AFM	Si	210 ± 20 zJ	190 ± 35 zJ		[25]
WSe_2_ (air)	FM‐AFM	Si	225 ± 30 zJ	220 ± 10 zJ		[25]
Silver	JC‐AFM	Si_3_N_4_	373 ± 89 zJ	240 zJ (vacuum); 139 zJ (water)	290 zJ	[24]
ZnO (air)	Bimodal AFM	Si	∼100 zJ			[106]
HZO‐L (air)	Bimodal AFM	Si	∼150 zJ			[106]
HZO‐M (air)	Bimodal AFM	Si	∼600±100 zJ			[106]
HZO‐H (air)	Bimodal AFM	Si	∼200 zJ			[106]

*Note*: Values from multiple AFM modes are compared with Lifshitz theory predictions, which provide a dielectric‐based theoretical benchmark. Agreement indicates consistency with electromagnetic theory, while differences reflect experimental conditions or model limitations. “PS” denotes polystyrene; other techniques are included where available.

Table 1 highlights the wide range of Hamaker constants measured across different material systems, spanning from ∼10 zJ for soft biological or molecular systems to several hundreds of zeptojoules for metals and layered materials. Overall, good agreement is observed between AFM‐derived values and Lifshitz theory predictions in many cases (e.g., SiO_2_, Al_2_O_3_, and MoS_2_), supporting the validity of AFM‐based approaches for quantifying vdW interactions at the nanoscale. However, noticeable variations exist depending on the AFM mode, tip material, and environmental conditions, reflecting sensitivity to experimental parameters and surface‐specific effects. The table also demonstrates general consistency across different AFM techniques, although discrepancies in some cases (e.g., mica, graphene systems) suggest the importance of calibration, modeling assumptions, and local heterogeneity. Collectively, these results confirm that AFM provides a versatile and reliable platform for probing vdW interactions, while also highlighting the need for careful interpretation when comparing across methods and theoretical models.

### 4.1 Quasi‐static Force‐Measurement Techniques

In the quasi‐static approach, AFM techniques are classified based on their approach to measuring vdW interactions and the Hamaker constant. The three primary quasi‐static methods identified include jump‐into‐contact (JC) [24], noncontact mode, and static contact mode. Each technique offers unique insights into vdW interactions, employing specific aspects of the cantilever deflection signal to probe material properties.

**FIGURE 4:**
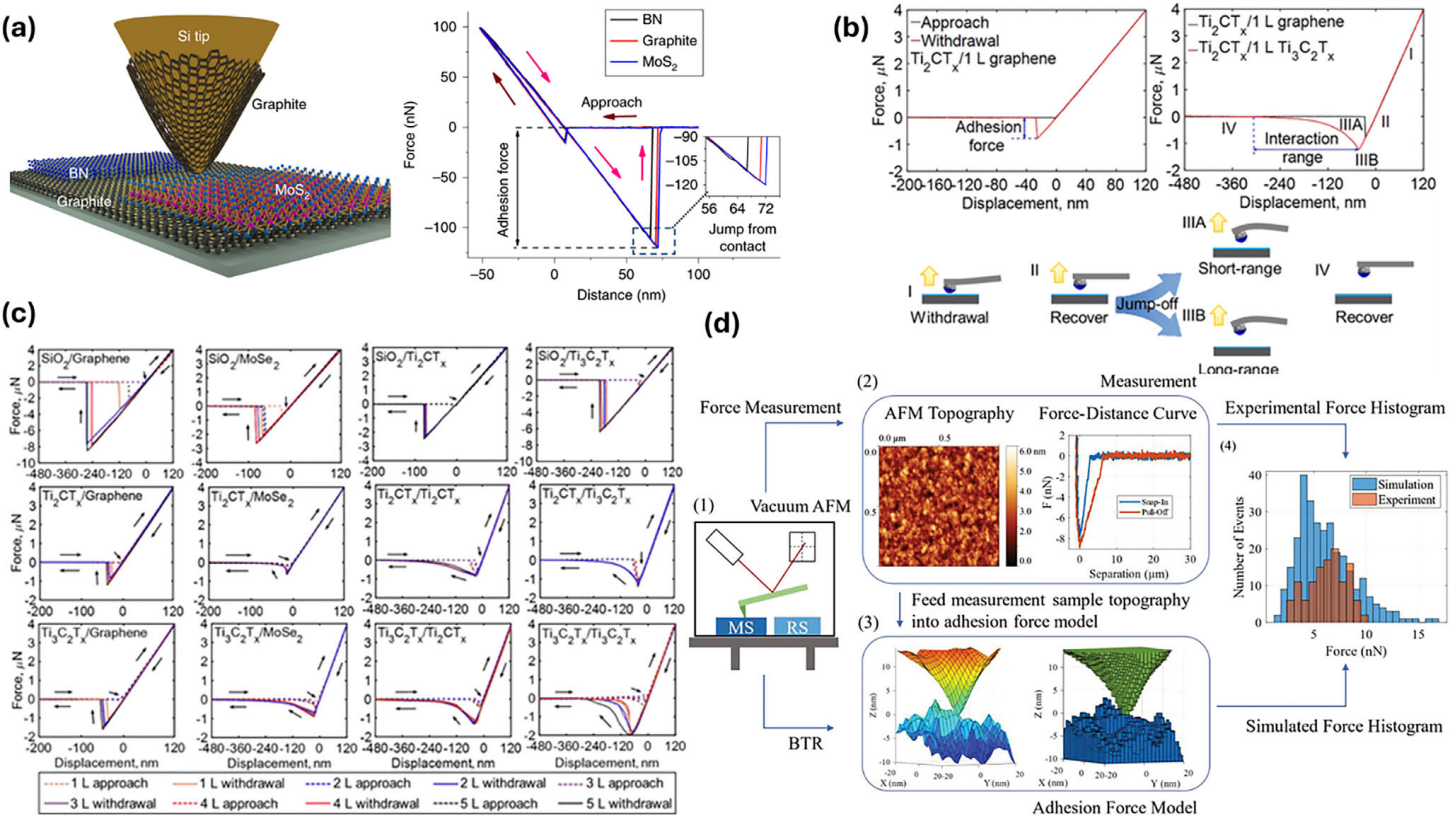
Quasi‐static AFM‐based characterization of vdW interactions and Hamaker constants. (a) Lattice structures of graphite, BN, and MoS_2_, highlighting surface atoms (with top‐ and bottom‐layer sulfur in MoS_2_ shown in blue and yellow) and schematic of an AFM tip wrapped with a thin graphite layer contacting BN–MoS_2_/graphite. The figure is reproduced with permission from reference [108]. Copyright 2019, Springer Nature. (b) Force–displacement curves measured during the AFM probe approach and withdrawal for Ti_2_CT*_x_*/graphene and Ti_2_CT*_x_*/Ti_3_C_2_T*_x_*, alongside schematics, illustrating tip‐bending stages. The figure is reproduced with permission from reference [111]. Copyright 2021, American Chemical Society. (c) Approach and withdrawal curves for probes coated with different materials and varying substrate layer numbers. The figure is reproduced with permission from reference [111] 2021, American Chemical Society. (d) Workflow of AFM‐based vdW force measurements (BTR technique), including surface topography scans, force–distance mapping, vdW modeling, and Hamaker constant extraction. The figure is reproduced with permission from reference [112]. Copyright 2023, John Wiley & Sons Inc.

The JC technique utilizes the “jump‐into‐contact” phenomenon, where the AFM cantilever experiences an instability and rapidly moves toward the sample surface as vdW forces overcome the cantilever's restoring force. The JC method assumes vdW‐dominated interactions and ideal cantilever behavior. By measuring the deflection of the cantilever at this JC point, Das et al. [24] could determine interaction parameters such as the Hamaker constant. They applied their method to determine the Hamaker constant of mica, silver, and SiO_2_ (see Table 1). JC is advantageous for hard samples, as it minimizes tip–sample deformation, providing a clearer distance measure for quantifying vdW forces accurately. This method emphasizes the role of the cantilever's spring constant and tip radius in determining the interaction strength, offering a straightforward approach to vdW measurements. Using the same approach in ultrahigh vacuum, Li et al. [108] examined vdW interactions in 2D heterostructures, focusing on graphite, BN, and MoS_2_ (Figure 4a). A graphite‐wrapped AFM tip (∼10‐nm thick) is used under high vacuum (∼1 × 10^−^^7^ torr) to measure these interactions without water interference. Layered BN and MoS_2_ flakes on graphite allow simultaneous analysis, revealing critical adhesion pressures similar to graphite–graphite adhesion, aligning with Lifshitz theory. MoS_2_ consistently shows a stronger attraction to graphite than BN, while conventional DFT‐based models struggle to predict these vdW interactions. Yu and Dai [109] showed that in AFM measurements on deformable (linearly elastic) substrates, vdW attraction can induce precontact surface deformation, effectively reducing the tip–sample separation and triggering a premature JC compared to rigid‐surface models. This is typically analyzed within a quasi‐static AFM approach, where the cantilever–sample system is treated as a balance of elastic‐restoring forces and long‐range vdW attraction, with analytical solutions derived for different substrate thickness limits. The study highlights that surface compliance can significantly bias force–distance interpretation, meaning that standard rigid‐body vdW models may overestimate the true separation at instability and thus affect the quantitative extraction of Hamaker constants in soft and biological materials. For deformable interfaces in liquid environments, the work on JC for elastic substrates [110] showed that AFM instability is governed by a coupled balance of vdW forces, elasticity, solid surface tension, and hydrodynamic lubrication effects. In this case, the AFM measurement is typically treated within a quasi‐static framework with dynamic corrections, where cantilever deflection and substrate deformation jointly determine the onset of instability. A key finding is that vdW attraction can deform soft gels or tissues beneath the probe, effectively reducing the gap and leading to earlier JC than predicted by rigid models, while solid surface tension modifies the deformation field, and elasticity remains essential for restoring long‐range mechanical stability. In addition, hydrodynamic drainage in liquid can delay contact, introducing rate‐dependent effects that further complicate the quantitative extraction of vdW interactions and Hamaker constants in soft‐matter systems.

In the noncontact approach, AFM detects attractive forces between the cantilever tip and the sample without physical contact, typically through frequency shift or deflection at the onset of attraction. The method often assumes that interactions are dominated by long‐range vdW forces and model the tip as an “effective sphere,” simplifying analysis by avoiding detailed tip geometry. However, this approximation can introduce uncertainty, particularly for sharp or irregular tips. In addition, electrostatic forces, hydration layers, and ambient conditions (e.g., humidity) may contribute to the measured signal, complicating interpretation. While the technique is well suited to soft or rough surfaces where contact methods are problematic, its quantitative accuracy depends on careful calibration, reliable knowledge of tip radius, and separation of vdW forces from other interactions. Using this method, Fronczak et al. [39] quantified the Hamaker constant of amorphous silica, polystyrene (PS), highly oriented pyrolytic graphite (HOPG), poly(methyl‐methacrylate) (PMMA), acrylonitrile–butadiene–styrene (ABS) polymer, and α‐Al_2_O_3_ (see Table 1).

**FIGURE 5:**
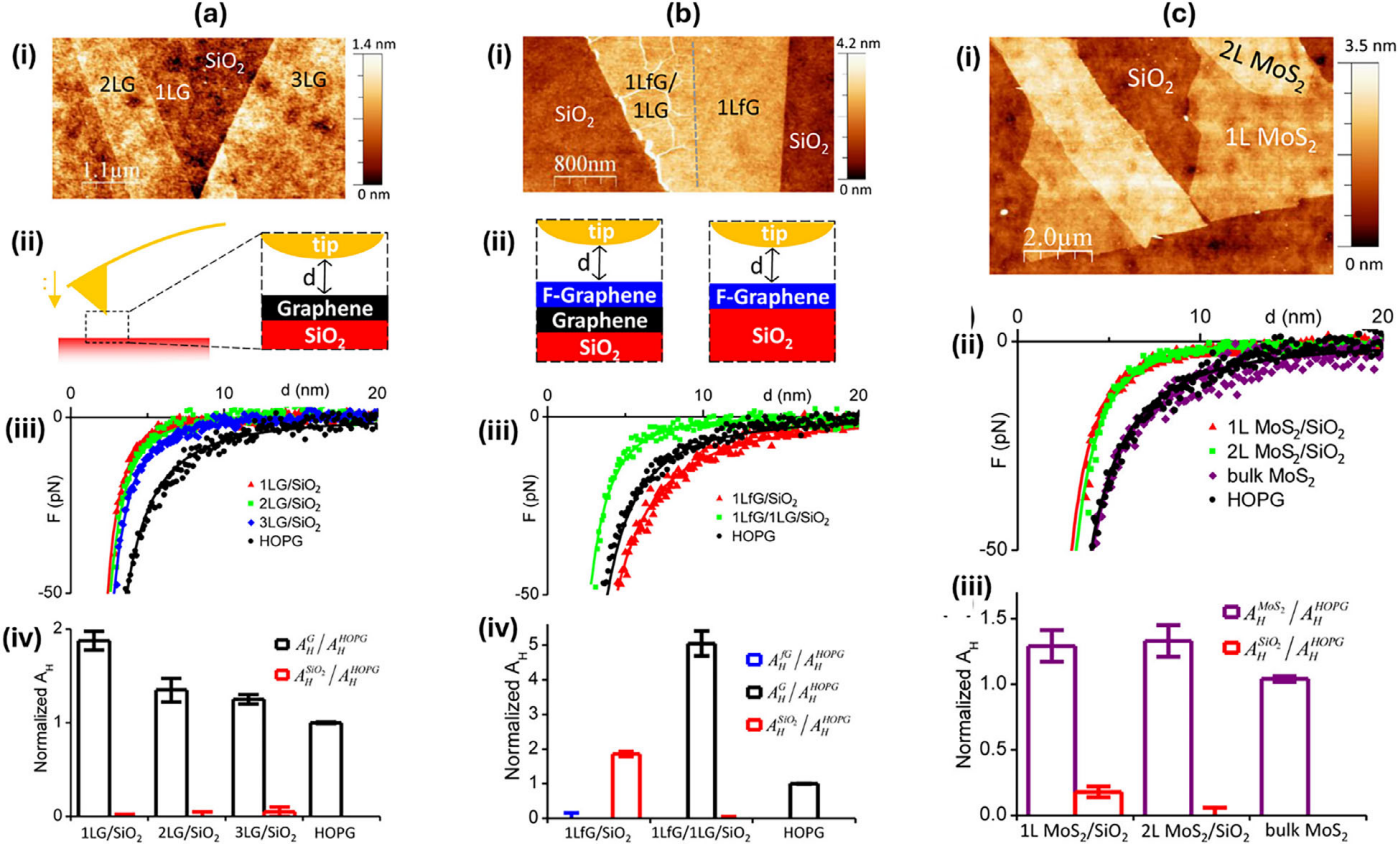
Quasi‐static AFM‐based studies of layered materials: (a) (i) AFM topography of graphene on SiO_2_ showing regions with different layer numbers (1LG, 2LG, and 3LG). (ii) Schematic of AFM force–distance measurement, where force *F* is recorded versus separation *d*. (iii) Measured *F*(*d*) curves for different graphene layers; dots: data, lines: fits; HOPG reference included. (iv) Extracted Hamaker coefficients for graphene/SiO_2_, normalized to HOPG. (b) (i) AFM topography of XeF_2_‐treated graphene on SiO_2_ showing single‐layer fluorinated graphene (1LfG) and bilayer 1LfG/1LG; dashed line marks their boundary. (ii) Schematic of AFM force measurements on 1LfG/SiO_2_ and 1LfG/1LG/SiO_2_. (iii) Representative F(d) curves acquired with a Si_3_N_4_ tip on both regions and HOPG (dots: data, lines: fits). (iv) Extracted Hamaker coefficients for fluorinated graphene, graphene, and SiO_2_, normalized to HOPG. (c) (i) AFM topography of MoS_2_ on SiO_2_ showing single‐ (1L) and double‐layer (2L) regions. (ii) Representative *F*(*d*) curves measured with a Si_3_N_4_ tip over 1L, 2L, and bulk MoS_2_, with HOPG reference (dots: data, lines: fits). (iii) Extracted Hamaker coefficients for MoS_2_ and SiO_2_, normalized to HOPG. The figures are reproduced with permission from reference [80]. Copyright 2014, American Chemical Society.

In contact mode, the AFM tip is brought into direct contact with the sample, and adhesion forces are measured as the cantilever pulls away. This approach is commonly used for mapping adhesion energies, particularly for nanoscale interfaces such as those between layered 2D materials. By measuring the pull‐off force required to break the contact, it could be possible to calculate adhesion energies and deduce vdW interactions, often applying models like the Rumpf model to quantify adhesion [111]. However, capillary forces, plastic deformation, and viscoelastic effects—particularly in soft or biological samples—can significantly influence the measured adhesion, leading to the overestimation of vdW contributions. Additionally, uncertainties in tip geometry, surface roughness, and model selection affect quantitative accuracy. While widely used for mapping adhesion at nanoscale interfaces, reliable extraction of vdW interactions requires careful calibration and consideration of competing forces. Li et al. [111] use MXene‐coated spherical AFM probes to measure adhesion between Ti_3_C_2_T*_x_*, Ti_2_CT*_x_*, graphene, and MoSe_2_ (Figure 4b‐c), utilizing the Rumpf model to derive adhesion values. They reported the highest adhesion for Ti_3_C_2_T*_x_* (1.23 J/m^2^) and lowest for MoSe_2_ (0.27 J/m^2^), with the longest interaction range in Ti_2_CT*_x_* interfaces. Ku et al. [112] applied vacuum AFM to analyze Si–tip interactions with Al_2_O_3_ and SiO_2_ on EUV photomasks, combining Hamaker theory with blind tip reconstruction (BTR) (Figure 4d). Hutter et al. [113] focused on precise AFM calibration for measuring vdW forces, observing transitions from nonretarded to retarded forces between a silicon nitride tip and mica. Tsoi et al. [80] investigated how graphene and MoS_2_ layers screen vdW forces from a silicon oxide substrate, examining effects of graphene layer count and doping (Figure 5). They find that vdW forces decrease with added graphene layers and hole doping, demonstrating that monolayers such as graphene and MoS_2_ can modify surface energy, with implications for vdW heterostructure design and nanomechanical devices. Furthermore, they quantified the interplay between the Hamaker constant of graphene and MoS_2_ layers with substrate (Table 1).

Chireux et al. [114] used both contact and FM‐AFM modes to study JC between deformable droplets, monitoring force and frequency shift during the approach. The results show that vdW attraction induces interface deformation, so the measured force–distance behavior is governed by a coupled vdW–capillarity balance rather than rigid‐body interaction. The analysis, based on a quasi‐static framework, identifies two regimes where the critical separation scales either with droplet size or becomes size independent, depending on whether deformation is geometry controlled or capillarity dominated. Overall, the study highlights that for liquid interfaces, vdW measurements are strongly influenced by surface tension‐driven deformation, which alters the effective interaction distance.

Krajina et al. [103] employed intrinsic friction analysis (IFA) to investigate the surface free energy of layered inorganic materials, focusing specifically on the Hamaker constant for graphene. IFA measures frictional forces during probe–sample interactions by analyzing the oscillatory motion of the probe tip, revealing interfacial energy and short‐range vdW contributions to surface free energy. Results indicate monolayer graphene has ∼0.4 times the energy of bulk graphite, with notable deviations at ∼3‐nm thickness (∼10 layers). Following pre‐characterization by micro‐Raman spectroscopy, IFA results show decreasing interfacial energy and vdW forces from monolayer to bulk, highlighting the impact of many‐body interactions and nonadditive effects in multilayer graphene. The IFA approach assumes that frictional dissipation is directly related to conservative surface forces; however, nonconservative effects such as viscoelasticity, plastic deformation, and energy dissipation mechanisms can complicate interpretation. In addition, sensitivity to tip condition, surface contamination, and calibration of lateral forces introduces uncertainty. Furthermore, recently Yu et al. [115] clarified the debated *vdW transparency* of graphene using colloidal AFM. They showed that vdW forces in graphene/SiO_2_ systems are not simply additive, with suspended monolayer graphene exhibiting a higher effective surface energy than when supported. Their pull‐in measurements revealed that 1–5 graphene layers screen roughly 15%–50% of the substrate's intrinsic vdW interaction, consistent with Lifshitz theory. These results demonstrate that even atomically thin layers significantly modulate intermolecular forces through nonadditive and substrate‐dependent effects. Similarly, Wang et al. [116] has provided direct experimental evidence for the *many‐body* nature of vdW interactions in multilayer structures using the quasi‐static AFM technique. By exploiting the atomic‐scale separation between an AFM tip and graphene‐supported substrates, researchers verified that substrate contributions to adhesion cannot be captured by simple pairwise dispersion models, which significantly overestimate the interaction. In contrast, MBD theory accurately describes the measured forces, revealing strong nonadditive effects governed by the spectrum of charge‐density fluctuations. Substrate‐dependent vdW interactions can be tuned through doping [117] and intercalation in layered materials [118]. Electronic modification (e.g., charge transfer in graphene) alters the spatial extent of π‐orbitals, changing adsorption energies and thus vdW binding strength. Similarly, intercalation in graphene/SiC systems significantly modifies surface structure, thermal expansion, electron–phonon coupling, and the effective interaction potential. These studies show that vdW interactions in 2D systems are not intrinsic constants but can be engineered via substrate and interface control. These insights show that vdW interactions in 2D material systems can be actively modulated, with implications for MEMS technologies and surface molecular assembly.

### 4.2 Dynamic Force‐Measurement Techniques

Dynamic force‐measurement techniques can be classified mainly into: amplitude‐modulation spectroscopy [99, 104, 119], frequency‐modulation spectroscopy [23, 120], peak force tapping (PFT) mode [101], phase imaging [79], torsional force microscopy (TFM) [121], and noncontact *q*‐Plus tuning fork frequency modulation [31, 122].

AM‐AFM, or tapping mode, operates by driving the cantilever near its resonance frequency and monitoring changes in oscillation amplitude and phase during intermittent tip–sample contact.

**FIGURE 6:**
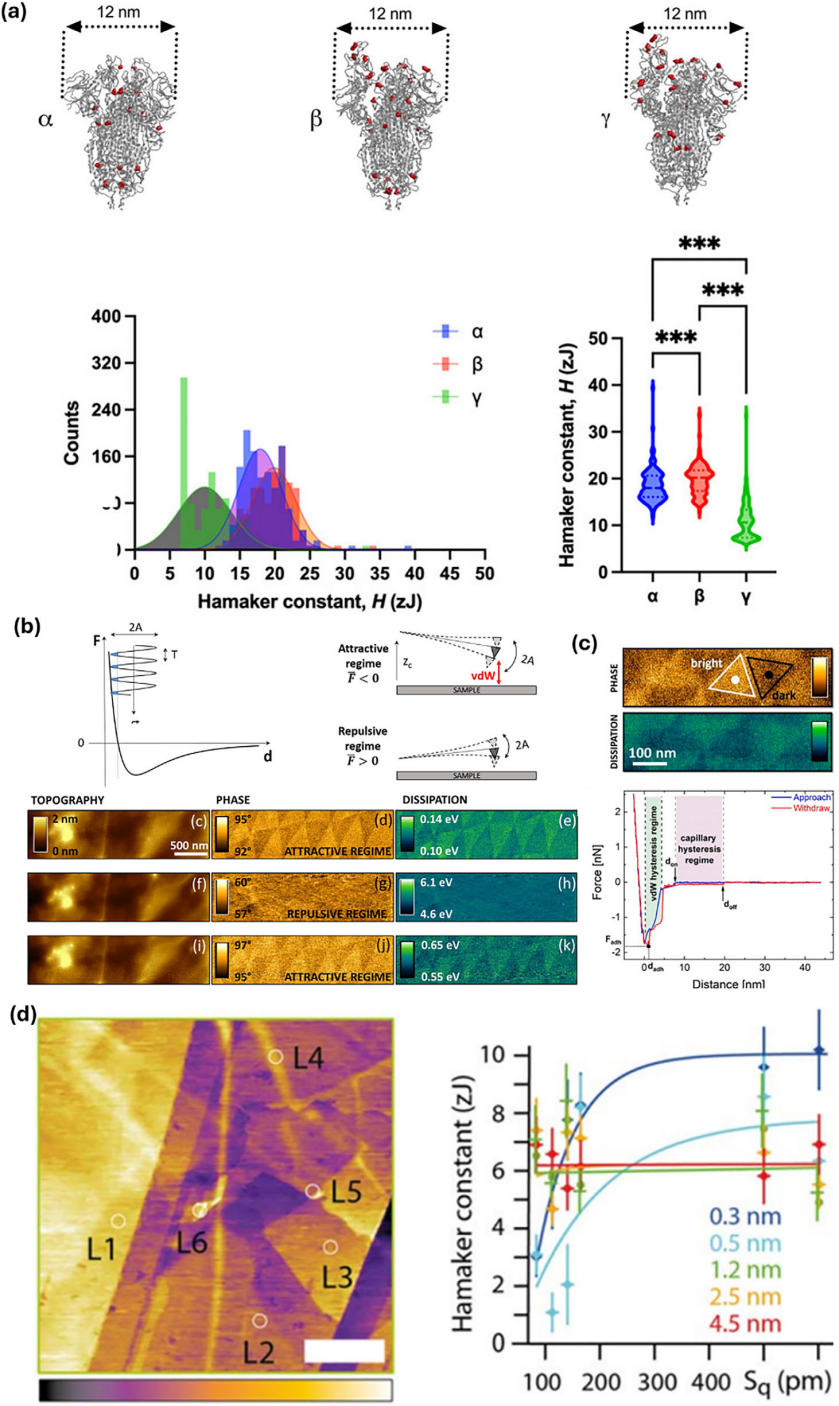
Dynamics AFM‐based characterization of vdW interactions and Hamaker constants. (a) Variations of spike proteins and corresponding changes in the Hamaker constant, with direct comparison of α‐, β‐, and γ‐proteins. The figure is reproduced with permission from reference [119]. Copyright 2023, John Wiley & Sons Inc. (b) Force–regime‐dependent tapping‐mode AFM imaging of Moiré contrast in twisted hBN. The top left is the Lennard–Jones (LJ) force–distance (*F*–*d*) plot illustrating attractive and repulsive regimes, with AFM tip oscillations mapped onto the LJ curve to highlight interactions within each oscillation period. The top right is the schematics of vdW‐related attractive regime (AR) and repulsive regime (RR), showing cantilever deflection and average position. The bottom figure is the topography, phase, and dissipation maps in AR (φ > 90°), revealing clear Moiré patterns, while in RR (φ < 90°) the contrast is suppressed; restoration of AR recovers the Moiré superlattice. The figure is reproduced with permission from reference [79]. Copyright 2022, American Chemical Society. (c) AFM phase and dissipation maps with bright/dark triangular Moiré domains; *n* = 300 *F*–*d* curves acquired at marked points reveal nanoscale water bridge hysteresis and adhesion force (*F*_adh_). The shaded regions highlight the two hysteresis regimes where the approach and withdraw curves do not overlap. The figure is reproduced with permission from reference [79]. Copyright 2022, American Chemical Society.  (d) Effect of nanoscale surface roughness on Hamaker constant at the HOPG–water interface, showing roughness‐dependent behavior at small oscillation amplitudes and convergence at larger amplitudes. At small oscillation amplitudes (<1 nm), Hamaker constant varies with the sample roughness. This is no longer the case for larger amplitude where Hamaker constant appears roughness independent due to the local features being averaged in the measurements. The figure is reproduced with permission from reference [23]. Copyright 2023, The Institute of Physics.

AM‐AFM has been used to characterize surfaces through a combination of raster scanning in tapping‐mode (AM‐AFM [107]) and dynamic‐force spectroscopy [99, 107, 119]. The tapping‐mode raster scanning allows noninvasive, high‐speed mapping of surface features, while force spectroscopy gathers detailed amplitude and phase profiles and reconstruct the interaction forces between the probe and sample [123, 124, 125]. This approach enables the measurement of multiple force profiles and effectively capturing data on both heterogeneous and atomically flat domains. By analyzing adhesion forces and nanometer‐scale indentation properties, the studies highlight the capabilities of AFM for distinguishing vdW terminations and mechanical properties of nanoscale materials. This approach assumes stable oscillation and a direct relation between amplitude/phase and conservative forces such as vdW interactions. However, energy dissipation, nonlinear dynamics, and cross talk with nonconservative forces can complicate interpretation. The findings across these studies demonstrate AM‐AFM's capability of analyzing diverse materials. AM‐AFM distinguished heterogeneous from atomically flat domains on 2D SnTiS_3_ surfaces, correlating adhesion force with vdW interactions and enabling the determination of Young's modulus at nanoscales [107]. Payam et al. [99] introduced an analytical model for quantifying viscoelastic properties and the Hamaker constant, validated through simulations and yielding consistent experimental results on LDPE and PS samples (Table 1), establishing the method's robustness for various materials. Moreover, Payam et al. [119] used AM‐AFM to quantify Hamaker constants for viral protein variants, highlighting distinct vdW interaction strengths among proteins (Figure 6a).

Furthermore, Chiodini et al. [79] explored the effect of twisting layered materials, particularly twisted hexagonal boron nitride (t‐hBN), on vdW interactions using tapping‐mode AFM phase imaging (Figure 6b,c). The technique visualizes the modulation of the vdW potential in the Moiré superlattice and reveals that vdW dissipation is influenced by the Debye force between the AFM tip and the material's dipoles, with modulation occurring at the surface aligned with stacking domains. The research demonstrates the advantages of tapping‐mode AFM over electric force microscopy for local adhesion engineering, nanoparticle deposition, and electrostatically patterned surfaces, highlighting the potential for manipulating vdW forces to engineer surface properties and develop advanced materials in nanotechnology.

FM‐AFM measures weak nanoscale forces by tracking shifts in the resonance frequency of an oscillating cantilever as it interacts with the sample; the cantilever is driven at resonance with constant amplitude, and tip–sample force gradients induce measurable frequency changes. This method is particularly useful for quantifying vdW forces, the Hamaker constant, and Young's modulus. It relies on precise frequency changes to determine these parameters, with some studies refining FM‐AFM by adjusting oscillation amplitudes to target different interaction scales, from sub‐nanometer defects to larger homogeneous regions. This technique simplifies data interpretation by relating frequency shifts directly to interaction forces, avoiding the complexity of multi‐harmonic deconvolutions. For example, Cafolla et al. [23] demonstrated its application in measuring vdW interactions in 2D materials and polymers, providing accurate Hamaker constant values consistent with theoretical models (Table 1) and showcasing its utility in areas such as energy storage and coatings. They have measured the Hamaker constant of HOPG in water, revealing the Hamaker constant of various surface features such as step edges, terraces, point‐like defects, and other singularities (Figure 6d).

**FIGURE 7:**
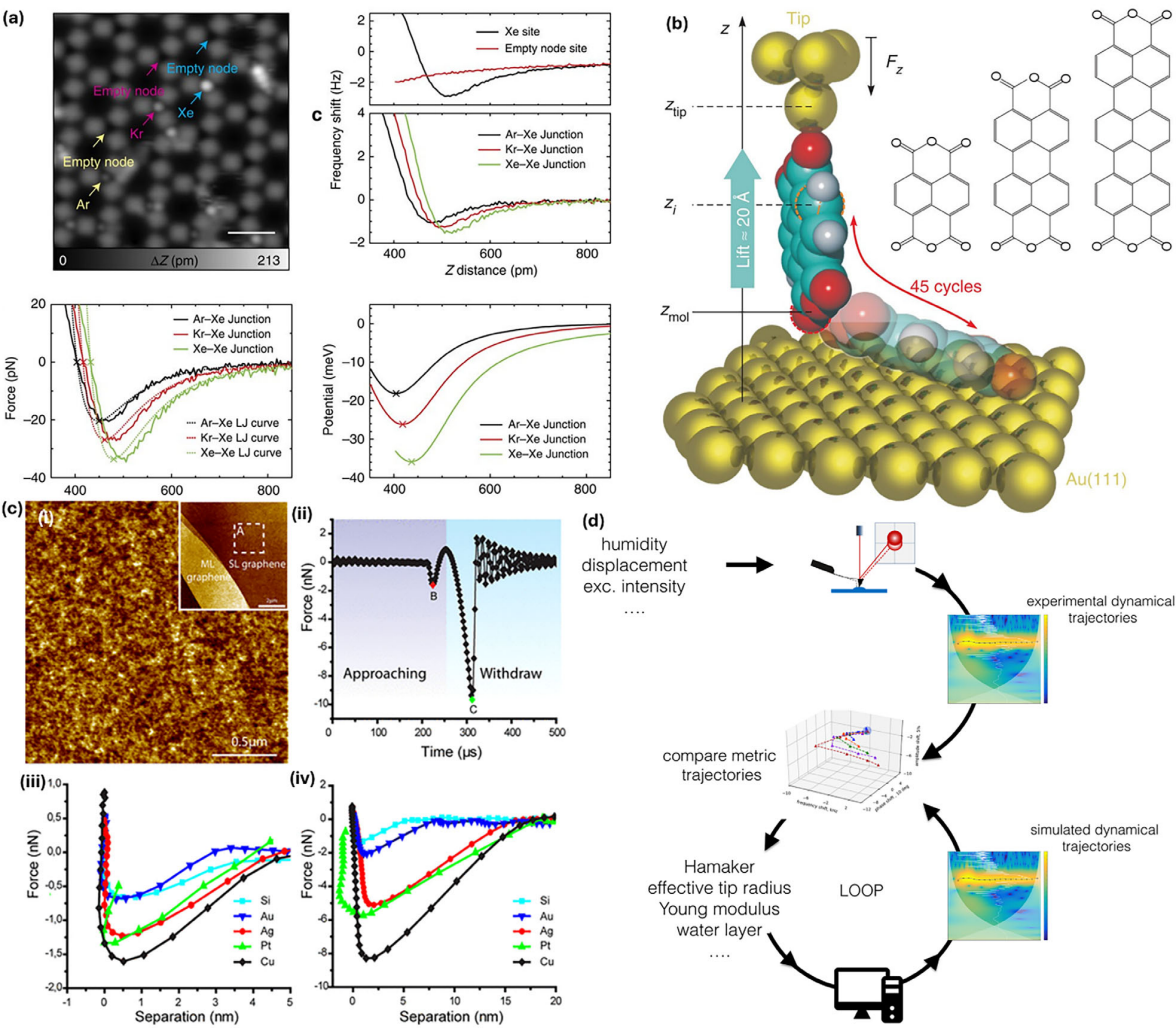
Dynamics AFM‐measurements of vdW force and Hamaker constant. (a) STM and NC‐AFM (FM‐AFM) measurements of rare‐gas–mediated interactions, including frequency shift curves, LJ fits, and potential energy profiles. Frequency shift curves taken at the equivalent node sites with and without a Xe atom, measured with a Xe‐functionalized tip. Molecular manipulation experiments probing force gradients during repeated lift–detach cycles of organic molecules. The figure is reproduced with permission from reference [120]. Copyright 2016, Springer Nature. (b) Schematic illustration of the experiment probing the nonadditivity of molecule–surface vdW potentials. In each experiment, a single isolated molecule is contacted at one of its reactive carboxylic oxygen atoms, detached from the surface, lifted by ∼2 nm, and subsequently returned to the adsorbed state. This cycle is repeated up to 45 times while continuously recording the force gradient (*dFz*/*dz*). The tip–surface distance (*z*_tip_), molecule–surface distance (*z*_mol_), and atomic distances (*z_i_*) relative to the surface are indicated. The chemical structures of the three investigated molecules are shown on the right: NTCDA, PTCDA, and TTCDA (left to right). The figure is reproduced with permission from reference [31]. Copyright 2014, Springer Nature. (c) Graphene morphology and interaction forces between a metal‐coated AFM probe and graphene. (i) AFM morphology image of graphene; (ii) representative force–time curve acquired with a Cu‐coated probe, where red (B) and green (C) markers denote adhesion forces during approach and withdrawal, respectively; (iii) force–separation curve obtained from the approach process (*F*_app_); and (iv) force–separation curve obtained from the withdrawal process (*F_w_*). The figure is reproduced with permission from reference [101]. Copyright 2013, American Chemical Society. (d) Schematic of the iterative process used to extract multiple physical parameters from tip–sample interactions [105]. Experimental cantilever traces under varying excitation intensities are wavelet transformed to generate metric trajectories, which are then compared with simulated trajectories obtained by systematically varying parameters (e.g., water layer height, elastic modulus, tip radius, and Hamaker constant). Minimization of error between experiment and simulation yields accurate multi‐physical parameters of the contact interaction. The figure is reproduced with permission from reference [105]. Copyright 2019, Springer Nature.

Kawai et al. [120] focused on noble gas atom pairs, measuring their vdW forces with high sensitivity and showing that Ar–Xe, Kr–Xe, and Xe–Xe interactions exhibit covalent characteristics, challenging traditional atomic interaction models (Figure 7a). These studies highlight FM‐AFM's versatility in quantifying interactions across diverse materials and its potential applications in nanotechnology, molecular binding, and material design. By employing FM‐AFM with a *q*‐Plus sensor (tuning fork), where one prong is fixed and the other carries a sharp tip oscillating at its resonance frequency, and frequency shifts induced by tip–sample force gradients are detected via the piezoelectric response, this setup enables highly sensitive and precise measurement of nanoscale interactions, allowing the investigation of weak vdW forces with exceptional resolution. This is crucial for studying the behavior of single molecules and their interactions with substrates. Schuler et al. [122] used this technique to study the adsorption geometry of π‐conjugated molecules on a Cu(1 1 1) substrate, achieving atomic resolution in lateral position, height differences, and molecular tilt. The results revealed how small structural changes in olympicenes alter adsorption, providing insights into vdW interactions at the molecular level under ultrahigh vacuum and low‐temperature conditions.

Wagner et al. [31] focused on vdW forces between π‐conjugated poly‐naphthalene derivatives and an Au(1 1 1) surface (Figure 7b), revealing a super linear increase in vdW interaction with molecular size due to electron deconfinement. These findings challenge conventional models and underscore the need for advanced theoretical approaches to account for nonadditive vdW contributions. The study confirmed that vdW forces are primarily influenced by molecule–surface interactions, with minimal impact from electrostatic effects or molecular orientation.

PFT mode is a dynamic AFM technique where the tip oscillates with controlled amplitude, making soft contact with the surface at the peak of each oscillation to measure interaction forces, adhesion, and stiffness with high precision. This mode provides accurate force–displacement curves and high‐resolution imaging while minimizing surface damage. Lazar et al. [101] employed the PFT technique to study the interaction between metal‐coated AFM tips and graphene. By quantifying the adhesion and peeling forces for various metals (Cu, Ag, Au, Pt, and Si) (Figure 7c), the research showed that copper had the strongest affinity for graphene. These measurements, taken at microsecond timescales, were corroborated by DFT calculations, including vdW effects, enhancing the understanding of metal–graphene interactions and electronic properties, with implications for advanced electronic device development.

Pendharkar et al. [121] introduced TFM, an AFM‐based technique operating in a dynamic torsional resonance mode with contact feedback control, where the cantilever is actively driven at its torsional resonance while maintaining a constant normal force on the surface. The torsional response, governed by dynamic frictional interactions, enables direct imaging of both atomic lattices and moiré superlattices in 2D heterostructures (e.g., graphene and hBN) under ambient conditions without electrical bias. This approach provides a powerful route to map structural variations such as twist angle and strain through friction‐sensitive contrast, highlighting how lateral force channels in AFM can access subsurface and interfacial structural information beyond conventional topographic or vertical force measurements. The few‐cycle regime AFM (FR‐AFM) technique [105] uses wavelet analysis to measure the transient response of a cantilever under impulsive excitation, offering enhanced sensitivity compared to traditional AFM methods. It delivers high‐bandwidth pulse‐like excitations and time‐localizes spectral components, enabling precise measurements of parameters such as the Hamaker constant, sample elasticity, and water layer height (Figure 7d). FR‐AFM significantly improves accuracy by analyzing transient data and comparing it with simulated traces, providing better sensitivity in determining key metrics like amplitude, frequency shifts, and phase lags. Automation with machine learning could further optimize parameter fitting in AFM applications.

Sub‐resonance tapping (SRT) [126, 127] is another advanced AFM scanning mode where the cantilever oscillates at a frequency significantly below its natural resonance. This technique, also known as ring‐mode AFM [127], allows for high‐resolution imaging and simultaneous mapping of multiple nanomechanical properties like adhesion and stiffness by analyzing the cantilever's behavior after each rapid, short tip–sample contact. Doukukin et al. [127] have applied this technique to measure the adhesion force of fixed human epithelial cells, corneocyte skin flakes, and polymers used for bioimplants.

Recently, Han et al. [128] introduced a light‐modulated vdW force microscopy approach based on conventional tapping mode, where the cantilever is driven near resonance while an external optical field is used to modulate the tip–sample interaction. This optical excitation induces a material‐dependent modulation of the vdW force, providing an additional contrast channel beyond conventional topography or phase signals. The key advance is that vdW interactions become dynamically tunable and composition‐sensitive under light excitation, enabling the discrimination of heterogeneous phases with sub‐10‐nm spatial resolution. This work highlights an emerging route in AFM where external stimuli (here optical fields) are used to enhance the chemical sensitivity of otherwise geometry‐dominated vdW‐based measurements.

Overall, across AFM dynamic force methods, vdW interactions and Hamaker constants are obtained indirectly from amplitude, phase, frequency shift, or force–distance data, making results strongly model dependent. AM‐AFM assumes that amplitude and phase changes mainly reflect conservative vdW forces, but energy dissipation and nonlinear dynamics can introduce significant uncertainty. FM‐AFM offers improved sensitivity by linking frequency shifts to force gradients yet still depends on assumptions about tip geometry and separation of long‐ and short‐range forces. Contact and peak‐force methods rely on contact mechanics models (e.g., JKR/DMT), where tip shape, adhesion hysteresis, and surface compliance can affect accuracy. *q*‐Plus FM improves force resolution but is often restricted to idealized environments.

### 4.3 Multifrequency AFM Techniques

Recently, a growing number of studies have focused on multifrequency AFM approaches, particularly bimodal AFM, for measuring vdW interactions and Hamaker constants with improved spatial resolution and quantitative sensitivity. In the multifrequency method, the cantilever is simultaneously excited at its first (and optionally higher) eigenmodes, while monitoring amplitude, phase, and/or frequency response of each mode independently. The key assumption is that each eigenmode behaves as an independent harmonic oscillator with minimal intermodal coupling and that the tip–sample interaction can be linearized over small oscillation amplitudes. It is further assumed that the measured modal responses can be directly mapped onto conservative (vdW) and dissipative components through appropriate theoretical models. However, in practice, nonlinear coupling between modes, energy transfer, and tip–sample hysteresis can violate these assumptions, introducing cross talk and affecting the quantitative accuracy of extracted Hamaker constants and force gradients.

Rodríguez and García [129] described a technique using bimodal AM‐AFM to map surface composition noninvasively. By exciting the first two modes of a cantilever with a hemispherical tip, the first mode maps topography, and the second mode detects long‐range attractive forces, including vdW interactions, with sensitivities down to 10^−11^ N.

**FIGURE 8:**
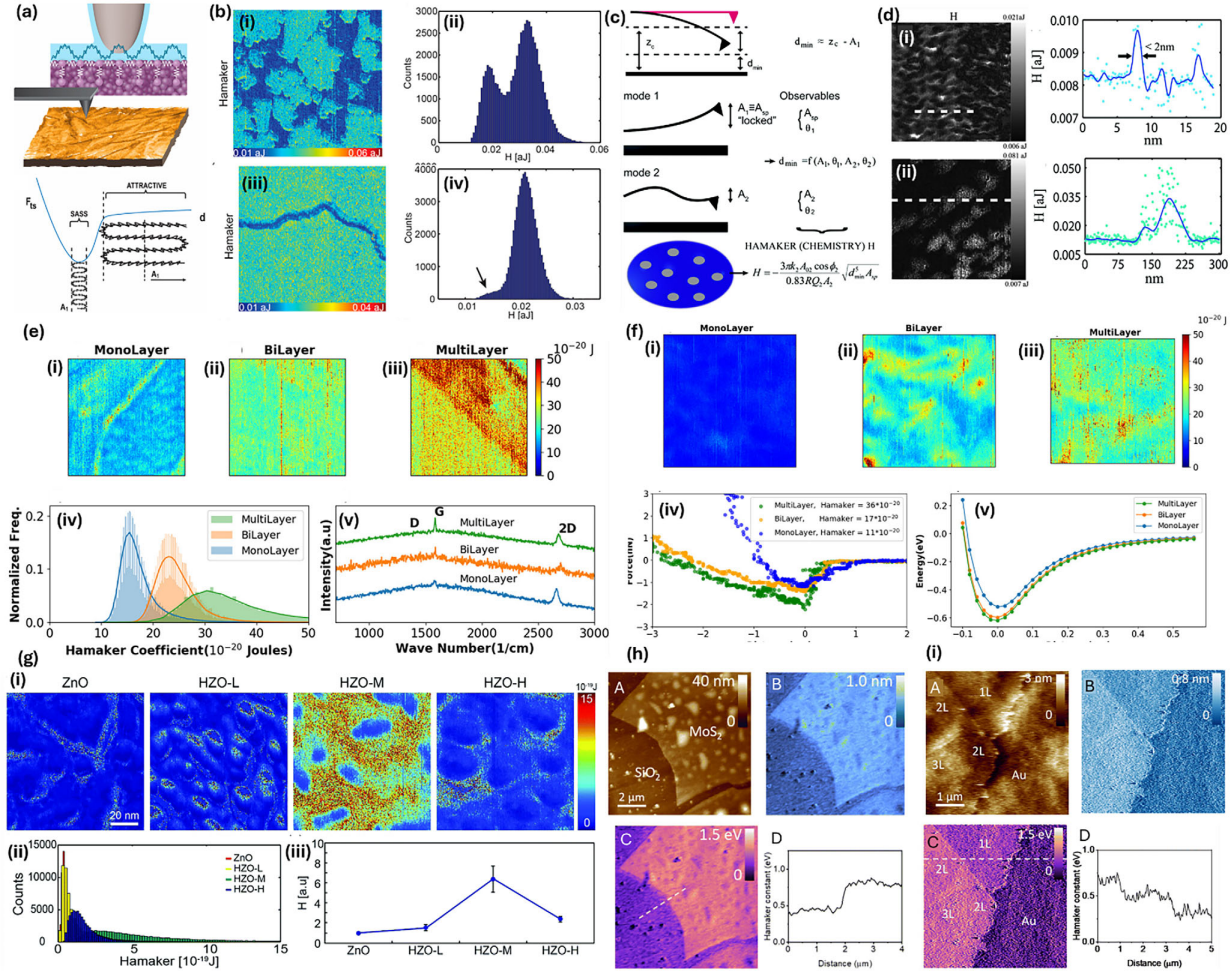
Multifrequency AFM‐based characterization of vdW interactions and Hamaker constants. (a) (i) Illustration of an AFM tip oscillating at small amplitudes and (ii) corresponding operating modes in the attractive and SASS regimes. The figure is reproduced with permission from reference [102] 2016, American Chemical Society. (b) Hamaker constant maps of (i) calcite (1 0 1) and (iii) DNA on mica, with (ii) histograms of the full data range; all images acquired in the attractive regime. The figure is reproduced with permission from reference [102] 2016, American Chemical Society. (c) Schematic of geometrical/operational parameters in bimodal AFM, where the Hamaker constant (H) is computed per pixel from four observables. The figure is reproduced with permission from reference [30] 2016, Royal Society of Chemistry. (d) Resulting H maps of (i) PFDA and (ii) calcite with cross‐sections along dashed lines. The figure is reproduced with permission from reference [30] 2016, Royal Society of Chemistry. (e) (i–iii) Hamaker coefficient maps (100 nm × 100 nm) of mono‐, bi‐, and multilayer graphene on Cu; (iv) coefficient distributions; (v) Raman spectra identifying mono‐, bi‐, and multilayer regions (raw data, no filtering; 532‐nm laser, spot radius ≈5 µm). The figure is reproduced with permission from reference [130] 2018, American Chemical Society. (f) (i–iii) Hamaker coefficient maps of free‐standing mono‐, bi‐, and multilayer graphene; (iv) reconstructed force profiles from AM‐AFM; (v) DFT‐predicted interaction energy versus the tip–graphene distance. The figure is reproduced with permission from reference [130] 2018, American Chemical Society. (g) Hamaker coefficient mapping of the ZnO and HZO films: (i) maps, (ii) distributions, and (iii) mean coefficient variations with Hf doping. The figure is reproduced with permission from reference [106] 2020, Royal Society of Chemistry. (h) Bimodal AFM of MoS_2_ on SiO_2_: (A) topography, (B) phase (first mode), (C) frequency shift (second mode), (D) minimum tip–sample distance, (E) Hamaker constant map, and (F) cross‐section showing variation from SiO_2_ to MoS_2_ [100]. The figures are reproduced from reference [100] 2023, Royal Society of Chemistry. (i) Bimodal AFM of MoS_2_ on Au: (A) topography of regions with 1L, 2L, and 3L domains, (B) phase, (C) frequency shift, (D) minimum distance, (E) Hamaker constant map, and (F) cross‐section showing two transitions consistent with three distinct surfaces. The figures are reproduced with permission from reference [100] 2023, Royal Society of Chemistry.

Lai et al. [102] have used bimodal AFM to enhance the resolution of measurements and create multiparameter maps of key physical properties such as stiffness, Hamaker constants, and adhesion forces. The method excites both AFM cantilever modes and monitors key parameters such as the amplitudes and phase of each mode. Materials such as calcite and dsDNA are used for expansion (Figure 8a,b). The study also introduces small amplitude small set‐point (SASS) AFM, improving resolution under water layers. Combining SASS with bimodal AFM refines measurements, revealing hidden information and advancing multidimensional AFM techniques for the scientific community.

Lai et al. [30] proposed a theory that uses four observables in bimodal AFM (amplitudes and phases of both first and second modes) to generate Hamaker constant maps, optimizing the operational parameters for high‐resolution imaging (Figure 8c). The methodology optimizes operational parameters to obtain Hamaker constant values consistent with Lifshitz theory, simplifying data acquisition for various cantilever–sample systems. While more complex systems may require model adjustments, the approach is widely applicable, enabling the quantification of chemistry in noncontact AFM imaging and providing valuable insights. They have applied their method on HOPG, mica, calcite, and PFDA (Table 1 and Figure 8d).

In their measurements, they show the importance of setting bimodal parameters to set the optimum minimum distance between the AFM tip and the sample surface for accurate measurements of the Hamaker constant.

Chiou et al. [130] focus on quantifying vdW forces in graphene grown by chemical vapor deposition (CVD). They use multifrequency AFM to assess the Hamaker coefficient, demonstrating that substrate choice significantly influences the vdW interactions measured (Figure 8e,f). Their approach also overcomes limitations of traditional force‐curve measurements.

Lai et al. [106] investigated the optoelectronic properties of hafnium‐doped zinc oxide (HZO) thin films, using AFM to measure vdW forces and surface energy. They examined the non‐monotonic optoelectronic behavior in HZO thin films, attributing it to competing mechanisms tied to crystal orientations during film growth. The (1 0 0) orientation, responsible for hafnium substitution, is hindered by increasing Hf concentration (Figure 8g). AFM, DFT, and high‐resolution transmission electron microscopy (HRTEM) data reveal that surface energy increases with Hf substitution, and crystal orientation shifts with higher Hf levels. The study suggests that an optimal balance of orientation and Hf concentration explain the non‐monotonic behavior, with future research potentially considering other factors like crystal shape.

Santos et al. [131] present a multifrequency AFM method that enhances the quantification of vdW interactions, using bimodal and trimodal techniques. The study highlights the effectiveness of the trimodal configuration (exciting the first three eigenfrequencies of cantilever and detecting the amplitude and phase of each mode) in improving sensitivity and robustness for weak‐force measurement, especially in nanofabrication and nanophotonic applications.

Santos et al. [132] explored the modeling of long‐range forces using power‐law expressions in multifrequency AFM. By applying these models, they show that the Hamaker constant can be quantified with high accuracy, aligning with theoretical frameworks such as Hamaker and Lifshitz theories.

Gisbert and Garcia [100] recently reported on the AM‐FM bimodal AFM technique for high‐resolution mapping of vdW forces and the Hamaker constant in 2D MoS_2_ materials. The method achieves exceptional spatial resolution and reveals differences in vdW interactions across various layers of MoS_2_, contributing to the understanding of material properties at the nanoscale (Figure 8h,i).

## 5 Measuring vdW Forces in a Liquid Environment

Quantifying vdW interactions in liquid environments presents a unique challenge for AFM, as the measured forces inevitably reflect the superposition of dispersion, electrostatic, and solvent‐mediated interactions. Disentangling these contributions is therefore essential for isolating medium‐dependent vdW forces and achieving reliable force interpretation at solid–liquid interfaces. A common approach is force–distance spectroscopy in a liquid cell, using a stiff cantilever and careful calibration of spring constant and tip geometry. Researchers often fit the attractive part of the curve with a vdW model, sometimes extended to account for the medium and tip shape, to separate vdW from other short‐range interactions [133, 134, 135].

At solid–liquid interfaces AFM separates medium‐dependent vdW interactions from electrostatic and hydration forces by exploiting their distinct spatial ranges, environmental sensitivities, and dynamic signatures. Early force–distance measurements in electrolytes demonstrated that long‐range electrostatic forces follow DLVO scaling and can be tuned or suppressed by ionic strength, while vdW interactions persist as a shorter‐range, medium‐dependent background governed by the dielectric properties of the intervening liquid [136]. In contrast, hydration (solvation) forces emerge at sub‐nanometer separations as oscillatory or exponentially decaying contributions [137], reflecting the discrete structuring of interfacial water, which cannot be captured by continuum vdW or electrostatic models. High‐resolution FM‐AFM and 3D force mapping have made this separation explicit by directly imaging hydration layers and correlating their spatial periodicity and dissipation signatures with molecular ordering rather than long‐range dispersion forces [138, 139]. Recent work demonstrates that AFM force measurements in liquid environments can disentangle vdW interactions from electrostatic and hydration forces by systematically tuning ionic strength and solvent conditions. The study shows that electrostatic forces follow DLVO behavior and are strongly screened with increasing salt concentration, while hydration forces emerge at sub‐nanometer distances as short‐range, oscillatory contributions. In contrast, the vdW interaction persists as a medium‐dependent background governed by the dielectric properties of the liquid. The results establish a practical framework for isolating dispersion forces at solid–liquid interfaces using controlled AFM force spectroscopy [140]. This work highlights also the importance of free‐energy calculations with high accuracy to estimate adsorption and derived forces, which is currently one of the key challenges in modeling vdW forces we discussed along our review and highlights it in Section 6 and Table 2. Furthermore, recently Ponce‐Gonzalez et al. [141] provided a practical protocol for measuring colloidal interactions using contact‐mode AFM, where a colloidal probe is brought into controlled approach–retraction with a functionalized surface to obtain force–distance curves. The study emphasizes the role of ionic strength and surface chemistry in modulating measured interactions and demonstrates how adhesion and long‐range forces can be extracted by fitting experimental data with an extended DLVO framework. This work highlights that, beyond vdW contributions, electrostatic double‐layer and hydrophobic interactions can dominate AFM force measurements in a solution, making accurate interpretation strongly dependent on surface preparation, environmental control, and model selection.

**TABLE 2:** Ten points focusing on future opportunities, open directions, and key unresolved problems in AFM‐based vdW force quantification.

No.	Theme/direction	Description and open challenge
1	Force disentanglement	Reliable separation of vdW from capillary, electrostatic, hydration, and dissipative forces under ambient/liquid conditions; need standardized protocols.
2	Tip geometry and evolution	Accurate correction for finite tip radius and wear; requires robust in situ tip characterization.
3	Multiscale theoretical integration	Bridging continuum, Lifshitz, and quantum many‐body models with measurable AFM observables.
4	Dielectric heterogeneity‐aware modeling	Incorporating spatial permittivity variations in layered and hybrid materials.
5	Environmental metrology	Controlling humidity, temperature, and gas effects for reproducible weak‐force measurements.
6	Forward‐model‐based interpretation	Linking multifrequency signals directly to interaction potentials, beyond inverse fitting.
7	Physics‐informed machine learning	Enabling uncertainty‐aware reconstruction and automated, physically consistent parameter extraction.
8	Advanced multifrequency AFM	Optimizing trimodal and higher modes for weak‐force sensitivity with minimal cross talk and noise.
9	Benchmark datasets	Creating open, standardized AFM datasets for validation and reproducibility.
10	Quantum metrology with AFM	Probing many‐body, nonadditive, and fluctuation‐driven interactions at interfaces.

## 6 Outlook

Recent advances in AFM, particularly multifrequency and bimodal AFM, have markedly enhanced the sensitivity and spatial resolution for probing vdW interactions and extracting Hamaker constants. Despite these improvements, accurately disentangling vdW forces from other competing tip–sample interactions remains challenging, especially under ambient conditions. A key obstacle is the presence of capillary forces arising from adsorbed moisture, which can dominate the interaction signal and mask vdW contributions. These effects can be minimized by conducting measurements under vacuum or within controlled environments, such as nitrogen‐filled chambers with residual oxygen levels below 0.2% [107], where water meniscus formation and environmental interferences are substantially reduced. Nevertheless, conducting AFM measurements in vacuum introduces its own set of challenges, such as maintaining a stable tip–sample interaction, tip contamination, and the need for specialized equipment, which may limit the accessibility and versatility of such measurements. In a nutshell, the challenges we identify for AFM‐based vdW quantification include topics such as capillary forces [142, 143], roughness [144], finite tip geometry [145], dielectric heterogeneity [146], and humid environmental conditions [147, 148]. Note that those topics are also in constant research and development and require a dedicated instrumentation perspective article.

In advancing the theoretical models underpinning AFM‐based vdW analysis, there is growing recognition that classical approximations often assuming idealized conditions such as atomically flat surfaces and perfectly smooth tip–sample contact are insufficient to capture the intrinsic complexity of real systems. While the Hamaker constant, traditionally derived from Lifshitz theory, provides a foundational means of quantifying vdW interactions, its accurate determination is complicated by nanoscale effects such as surface roughness, finite tip geometry, dielectric heterogeneity, and environmental fluctuations. To address these challenges, modern approaches increasingly integrate quantum mechanical frameworks, including MBD theory and perturbative treatments, which explicitly account for electron correlation, collective excitations, and quantum fluctuations that dominate at the nanoscale.

The motivation for these developments is rooted in their direct relevance to AFM experiments: precise measurement of vdW forces and Hamaker constants demand a theory that reflects not only macroscopic electrodynamics but also the quantum nature of matter–field interactions. By coupling refined theoretical treatments ranging from Lifshitz‐based continuum electrodynamics to quantum‐informed models such as MBD with high‐resolution AFM experiments, we can bridge the gap between quantum theory and measurable observables. This synergy not only improves the accuracy and reliability of AFM‐based force spectroscopy but also establishes AFM as a platform for probing fundamental quantum phenomena at material interfaces.

Such theoretical and experimental progress elevates AFM beyond a mere imaging or force‐measurement tool, positioning it as a frontier technique for nanoscale quantum metrology. This opens transformative opportunities in nanophotonics, quantum materials, and nanofabrication, where precise knowledge of quantum‐driven interactions is essential for the design of next‐generation devices and architectures.

Looking forward, there are numerous opportunities to advance the field of AFM‐based force measurements. One area of opportunity lies in the integration of machine learning and automation to further optimize the parameter fitting and data analysis in AFM experiments [149, 150, 151, 152]. Additionally, the ongoing development of trimodal and other higher order multifrequency AFM techniques offers promise for enhancing sensitivity and robustness, particularly in weak‐force measurements. The challenge of tip radius also warrants attention, as the radius influences both the force measurement sensitivity and the ability to quantify vdW forces accurately. Advances in tip fabrication, such as sharper and more uniform tips, could improve spatial resolution and accuracy in force measurements.

As these techniques continue to evolve, it is crucial to address the complex interplay between experimental conditions, theoretical models, and measurement precision. The future of the AFM‐based quantification of vdW interactions and other weak forces depends on overcoming these challenges, leading to more reliable and detailed insights into nanoscale materials and interactions.

Looking ahead, several key developments could substantially advance the field of AFM‐based vdW force quantification. A major priority is the systematic integration of AFM measurements with simulation and theoretical frameworks to improve interpretability and pinpoint the origins of specific interaction regimes. Continued progress toward a comprehensive theory of intermolecular interactions in complex and low‐dimensional materials remains essential, especially when coupled directly with multifrequency AFM observables. Forward modeling approaches that link AFM signals to underlying 2D material interactions would greatly simplify the interpretation of experimental force maps, while reanalyzing existing AFM databases could enable explicit reconstruction of theoretical scaling laws. Finally, unifying experimental results that probe vdW interactions across multiple length scales will be crucial for establishing a coherent, multiscale understanding of the physics governing layered materials and nanoscale interfaces. As a summary of this section, the main points focusing on future opportunities, open directions, and key unresolved problems in AFM‐based vdW force quantification (see Table 2).

## Author Contributions

**Amir Farokh Payam**: conceptualization, investigation, funding acquisition, Writing – original draft, methodology, visualization, Writing – review and editing, validation. **Horacio V. Guzman**: conceptualization, investigation, funding acquisition, writing – review and editing, visualization, validation. **Alexandre Tkatchenko**: conceptualization, methodology, funding acquisition, writing – review and editing, validation, supervision.

## Conflicts of Interest

The authors declare no conflicts of interest.

## Data Availability

The data that support the findings of this study are available from the corresponding author upon reasonable request.
